# Expanding Lipidomic Coverage in Multisegment Injection–Nonaqueous Capillary Electrophoresis–Mass Spectrometry via a Convenient and Quantitative Methylation Strategy

**DOI:** 10.1021/acs.analchem.3c02605

**Published:** 2023-11-22

**Authors:** Ritchie Ly, Lucas Christian Torres, Nicholas Ly, Philip Britz-McKibbin

**Affiliations:** Department of Chemistry and Chemical Biology, McMaster University, 1280 Main Street West, Hamilton, Ontario, Canada L8S 4M1

## Abstract

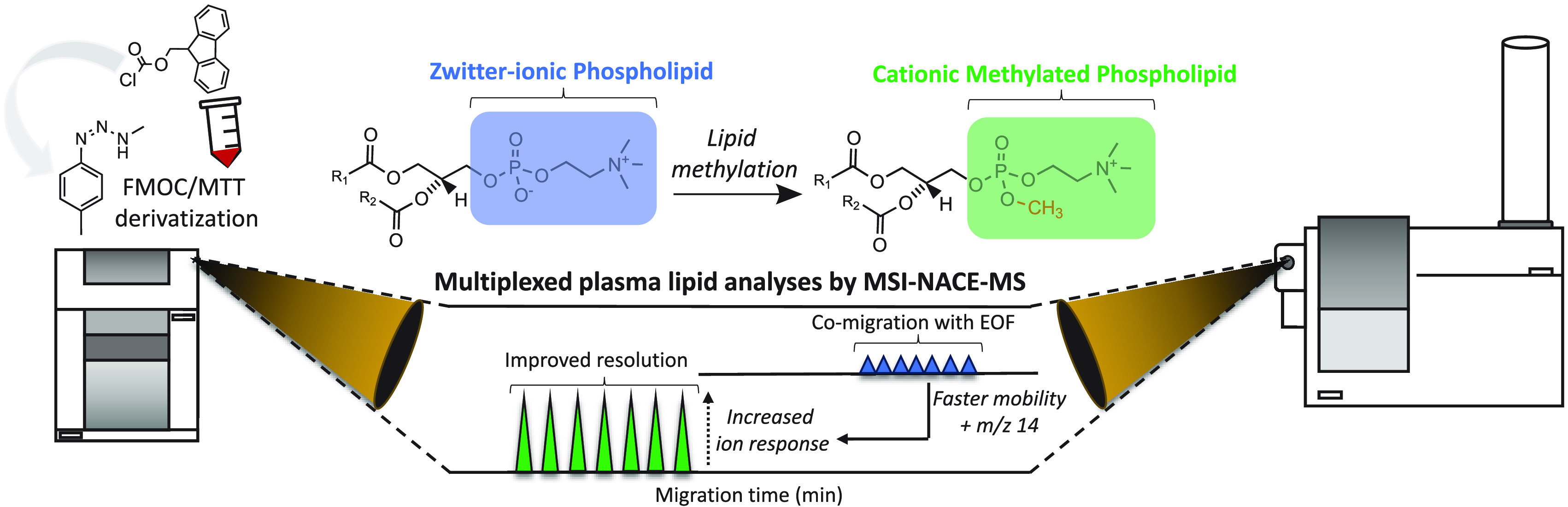

Orthogonal separation techniques coupled to high-resolution mass spectrometry are required for characterizing the human lipidome, given its inherent chemical and structural complexity. However, electrophoretic separations remain largely unrecognized in contemporary lipidomics research compared to established chromatographic and ion mobility methods. Herein, we introduce a novel derivatization protocol based on 3-methyl-1-*p*-tolyltriazene (MTT) as a safer alternative to diazomethane for quantitative phospholipid (PL) methylation (∼90%), which enables their rapid analysis by multisegment injection–nonaqueous capillary electrophoresis–mass spectrometry (MSI-NACE-MS). Isobaric interferences and ion suppression effects were minimized by performing an initial reaction using 9-fluorenylmethyoxycarbonyl chloride prior to MTT and a subsequent back extraction in hexane. This charge-switch derivatization strategy expands lipidome coverage when using MSI-NACE-MS under positive ion mode with improved resolution, greater sensitivity, and higher throughput (∼3.5 min/sample), notably for zwitterionic PLs that are analyzed as their cationic phosphate methyl esters. Our method was validated by analyzing methyl-*tert*-butyl ether extracts of reference human plasma, which enabled a direct comparison of 48 phosphatidylcholine and 27 sphingomyelin species previously reported in an interlaboratory lipidomics harmonization study. The potential for plasma PL quantification by MSI-NACE-MS via a serial dilution of NIST SRM-1950 was also demonstrated based on estimation of relative response factors using their reported consensus concentrations. Moreover, lipid identification was supported by modeling predictable changes in the electrophoretic mobility for cationic PLs in conjunction with MS/MS. Overall, this work offers a practical derivatization protocol to expand lipidome coverage in CE-MS beyond the analysis of hydrophilic/polar metabolites under aqueous buffer conditions.

## Introduction

The human lipidome comprises a vast number of lipid molecular species present in tissues, cells, exosomes, and biofluids, which are defined by their specific polar headgroup, chemical linkage, fatty acid carbon chain length, number of double bond equivalents, oxygenated fatty acyls, and regio/stereochemistry.^1,2^ As lipid homeostasis plays an important role in energy metabolism, membrane structure, and cell signaling, dysregulation in lipid metabolism has long been associated with inflammation and the etiology of cardiometabolic disorders, including obesity, type 2 diabetes, and cardiovascular and neurodegenerative diseases.^3,4^ Lipidomic studies have also gained traction in nutritional epidemiology as objective indicators of food exposures since essential dietary fats and fat-soluble vitamins relevant to human health^5^ are not accurately assessed from self-reports.^6^ For these reasons, new advances in untargeted lipid profiling by high-resolution mass spectrometry (MS)^7^ provide a hypothesis-generating approach for gaining new insights into complex disease mechanisms.^8^ However, several technical hurdles impede the progress in lipidomics, given the lack of chemical standards and reference MS/MS spectra that limit comparative quantitative reporting and the unambiguous identification of unknown lipids of clinical significance.^9^ Recent efforts have focused on developing consensus guidelines in lipid classification and annotation,^10,11^ using internal standards for data normalization,^12^ applying automated data processing with open-access software tools,^13,14^ as well as implementing standardized lipidomic protocols and interlaboratory ring trials using reference and quality control samples.^15−17^ Nevertheless, lipidomics workflows require careful method optimization to avoid bias and false discoveries depending on the specific biospecimen type and instrumental platform, including sample pretreatment protocols.^18^

Classical methods for lipid profiling of biological samples have relied on the analysis of esterified fatty acids from lipid hydrolysates using gas chromatography (GC)-MS.^19^ However, comprehensive analysis of intact phospholipids (PLs) was first achieved by MS when using soft ionization methods based on matrix-assisted laser desorption/ionization and electrospray ionization (ESI).^20^ Although shotgun lipidomics enables the direct analysis of lipid extracts by direct infusion (DI)-MS,^21^ high-efficiency separations are often needed to improve method selectivity while reducing ion suppression effects, isobaric interferences, and/or various other mass ambiguities.^22^ To date, liquid chromatography (LC)-MS remains the separation platform of choice in lipidomics.^23^ However, LC-MS protocols vary substantially in terms of operation conditions (e.g., column types, elution conditions, etc.) used to resolve different lipid classes primarily by reversed-phase, normal-phase, and/or hydrophilic interaction chromatography (HILIC).^24,25^ For instance, greater sample throughput, separation resolution and/or reproducibility can be achieved in reversed-phase LC-MS lipidomic analyses using core–shell particles,^26^ vacuum jacked columns,^27^ capillaries operated under ultrahigh pressure conditions,^28^ and via multidimensional separations.^29^ Alternatively, supercritical fluid chromatography-MS can resolve lipids that vary widely in their polarity with better robustness than HILIC-MS.^30^ Also, ion mobility-MS enables the ultrafast separation of PLs compared to chromatographic methods with adequate selectivity to generate a lipidome atlas.^31^ On the other hand, nonaqueous capillary electrophoresis–mass spectrometry (NACE-MS) is largely an unrecognized separation technique in lipidomics likely due to a paucity of published studies limited to certain ionic lipids, such as saturated fatty acids,^32^ lipid A isomers,^33^ and glycerophospholipids.^34,35^ Indeed, a lack of robust NACE-MS protocols, limited vendor support, and sparse method validation relative to existing chromatographic methods have deterred its use as a viable separation platform in untargeted lipid profiling.

Recently, we have introduced multisegment injection (MSI)-NACE-MS as a multiplexed separation platform for the quantitative determination of fatty acids from blood specimens,^6,36,37^ which can also resolve other classes of anionic lipids under negative ion mode detection, such as phosphatidic acids and phosphatidylinositols.^38^ A serial injection of seven or more samples within a single capillary allows for higher sample throughput^39^ together with temporal signal pattern recognition in ESI-MS^40^ for rigorous molecular feature selection and lipid authentication when performing nontargeted screening.^38^ However, separation resolution and selectivity are currently limited for phosphatidylcholines (PC) and other classes of zwitterionic lipids that migrate close to the electroosmotic flow (EOF). Precolumn chemical derivatization strategies have been developed to introduce or switch charge states on specific lipid classes to modify their chromatographic retention, reduce isobaric interferences, and improve ionization efficiency with lower detection limits in ESI-MS.^41^ For instance, Smith et al.^42−44^ have used diazomethane for charge inversion on modified cationic PLs via quantitative methylation. However, given the explosive and toxicity hazards of diazomethane that is generated *in situ*,^45^ safer methylating agents are required in routine MS-based lipidomic workflows without blast shields and other personal protective equipment. Herein, we introduce a novel two-step chemical derivatization strategy for the quantitative methylation of PLs based on 9-fluorenylmethyoxycarbonyl chloride (FMOC), followed by 3-methyl-1-*p*-tolyltriazene (MTT) that offers a practical way to expand lipidome coverage in MSI-NACE-MS. For the first time, we demonstrate that this procedure enables the rapid identification and quantification of PCs and sphingomyelins (SMs) as their cationic phosphate methyl esters, which was validated on a standard reference human plasma sample previously analyzed in an interlaboratory harmonization study.^15^

## Experimental Section

### Chemicals and Materials

Ultra LC-MS grade methanol, acetonitrile, water, and 2-propanol were used to prepare the sheath liquid and the background electrolyte (BGE). Ammonium formate, formic acid, 1,2-distearoyl-d70-*sn*-glycero-3-phosphocholine (PC 36:0[D70]), 1,2-dipalmitoyl-d62-*sn*-glycero-3-phosphocholine (PC 32:0[D62]), methyl-*tert*-butyl ether (MTBE), MTT, FMOC and all other chemical standards were purchased from Sigma-Aldrich Inc. (St. Louis, MO) unless otherwise stated. All lipid standards purchased were either as a powder or dissolved in solution (1:1) of chloroform and methanol. Stock solutions for lipids were then diluted in chloroform and methanol and stored at −80 °C prior to further use. The reference material from the National Institute of Standards and Technology (NIST) SRM-1950 pooled human plasma was purchased from the NIST (Gaithersburg, MD). While certified reference values for NIST SRM-1950 have been reported for several polar metabolites, plasma PLs measured in this study were compared to the median of mean concentrations reported for NIST SRM-1950 in an international study across 31 laboratories that adopted various LC-MS/MS lipidomic workflows. In this case, consensus plasma PL concentrations required measurements from a minimum of five laboratories having a sample coefficient of dispersion (COD) < 40%.^15^

### Plasma Lipid Extraction Using MTBE

Plasma samples and lipid calibrant solutions were extracted using a modified MTBE-based liquid extraction procedure previously described for fatty acids and anionic lipids using MSI-NACE-MS in negative ion mode.^36,38^ Briefly, 50 μL of an NIST SRM-1950 plasma aliquot was mixed with 100 μL of methanol containing PC 32:0[D62] as a recovery standard and shaken for 10 min. Then, 250 μL of MTBE was added, and the mixture was subjected to vigorous shaking for 10 min. To induce phase separation, 100 μL of deionized water was then added prior to centrifugation at 10 min at 4000*g*. Next, 200 μL of the lipid-rich MTBE upper layer was transferred into another vial and dried at room temperature using an Organomation MULTIVAP nitrogen evaporator (Berlin, MA). For underivatized lipids, dried plasma extracts were then reconstituted to a volume of 50 μL containing acetonitrile/isopropanol/water (70:20:10) with 10 mM ammonium formate containing internal standards PC 36:0[D70] (5 μM), benzyltriethylammonium chloride (BTA) (1 μM), and PC 32:0[D62] (5 μM) prior to analysis by MSI-NACE-MS.

### Chemical Derivatization of Zwitterionic Phospholipids Using FMOC and MTT

All plasma ether extracts and PL calibrants were subject to a two-step chemical labeling procedure using FMOC and MTT. In 2 mL amber glass vials, 100 μL of 0.85 mM FMOC in chloroform was added to dried ether plasma extracts, and the mixture was shaken vigorously for 5 min. Then, samples were blown down to dryness using nitrogen at room temperature prior to reconstitution in 50 μL of MTBE containing 450 mM MTT. Vials were next sealed with Teflon tape and vortexed for 30 s prior to derivatization at 60 °C for 60 min (unless otherwise stated). Afterward, 100 μL of methanol, 250 μL of hexane, and 200 μL of deionized water was added to back extract polar byproducts of the reaction (e.g., *p*-toluidine). After centrifugation for 10 min at 4000*g*, 200 μL of hexane as the supernatant was transferred out to a separate glass vial and then evaporated to dryness under nitrogen. Lastly, derivatized extracts were then reconstituted in 50 μL of acetonitrile/isopropanol/water (70:20:10) with 10 mM ammonium formate containing internal standards PC 36:0[D70] (5 μM), BTA (1 μM), and of PC 32:0[D62] (5 μM) prior to analysis by MSI-NACE-MS. Derivatization yields for methylated PLs from plasma extracts were calculated based on the integrated relative peak area (RPA) for each native (untreated) PL relative to PC 36:0[D70] as an internal standard using eq 1:
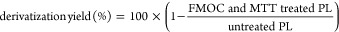
1

### CE-MS Instrumentation and Serial Injection Configuration

An Agilent 6230 time-of-flight (TOF) mass spectrometer with a coaxial sheath liquid electrospray (ESI) ionization source equipped with an Agilent G7100A CE unit was used for all experiments (Agilent Technologies Inc., Mississauga, ON, Canada). An Agilent 1260 Infinity isocratic pump and a 1260 Infinity degasser were utilized to deliver an 80:20 methanol:water with 0.1 vol % formic acid at a flow rate of 10 μL/min using a CE-MS coaxial sheath liquid interface kit. For mass correction in the real time, the reference ions purine and hexakis(2,2,3,3-tetrafluoropropoxy)phosphazine (HP-921) were spiked into the sheath liquid at 0.02 vol % to provide constant mass signals at *m*/*z* 121.0509 and 922.0098, which were utilized for monitoring ion suppression and/or enhancement effects. During sample introduction into the capillary, the nebulizer gas was turned off to prevent siphoning effects that may contribute to air bubbles and current errors upon voltage application.^36^ This was subsequently turned on at a low pressure of 4 psi (27.6 kPa) following voltage application, with the ion source operating at 300 °C with a drying gas of nitrogen that was delivered at 4 L/min. The TOF-MS was operated in a 2 GHz extended dynamic range under positive mode detection. A V_cap_ was set at 3500 V while the fragmentor was 120 V, the skimmer was 65 V, and the octopole rf was 750 V. All separations were performed using bare fused-silica capillaries with 50 μm internal diameter, a 360 μm outer diameter, and 100 cm total length (Polymicro Technologies Inc., AZ). A capillary window maker (MicroSolv, Leland, NC) was used to remove 7 mm of the polyimide coating on both ends of the capillary to prevent polyimide swelling with organic solvents in the background electrolyte (BGE) or aminolysis under alkaline nonaqueous buffer conditions.^46^ An applied voltage of 30 kV was used for CE separations at 25 °C together while using a forward pressure of 5 mbar (0.5 kPa). The BGE was 35 mM ammonium formate in 70 vol % acetonitrile, 15 vol % methanol, 10 vol % water, and 5 vol % isopropanol with an apparent pH of 2.3 adjusted with the addition of formic acid. Derivatized plasma extracts and lipid standards were introduced in-capillary hydrodynamically at 50 mbar (5 kPa) alternating between 5 s for each sample plug and 40 s for the BGE spacer plug for a total of seven discrete samples analyzed within a single run.^38^ Prior to the first use, capillaries were conditioned by flushing at 950 mbar (95 kPa) with methanol, 0.1 M sodium hydroxide, deionized water, and BGE sequentially for 15 min each. The BGE and sheath liquid were degassed prior to use. For analysis of NIST SRM-1950 by MSI-NACE-MS in negative ion mode to verify acidic lipids not amenable by the FMOC/MTT labeling, an alkaline BGE with the same organic solvent composition was used, but with ammonium acetate and ammonium hydroxide as the BGE and pH modifier respectively as described elswhere.^36^ In this case, the same MTBE extraction protocol was applied for the direct analysis of fatty acids and anionic lipids, but the extract was concentrated 2-fold without FMOC/MTT chemical derivatization. Plasma PLs were annotated by MSI-NACE-MS based on their sum composition, mass error, and relative migration times (RMTs) or apparent electrophoretic mobilities (Tables S1 and S2) with selected PLs from NIST SRM-1950 ether extracts further characterized by MS/MS for confirmation of molecular PC and SM species.

## Results and Discussion

### Separation Performance Enhancement after Phospholipid Methylation

**Figure 1:**
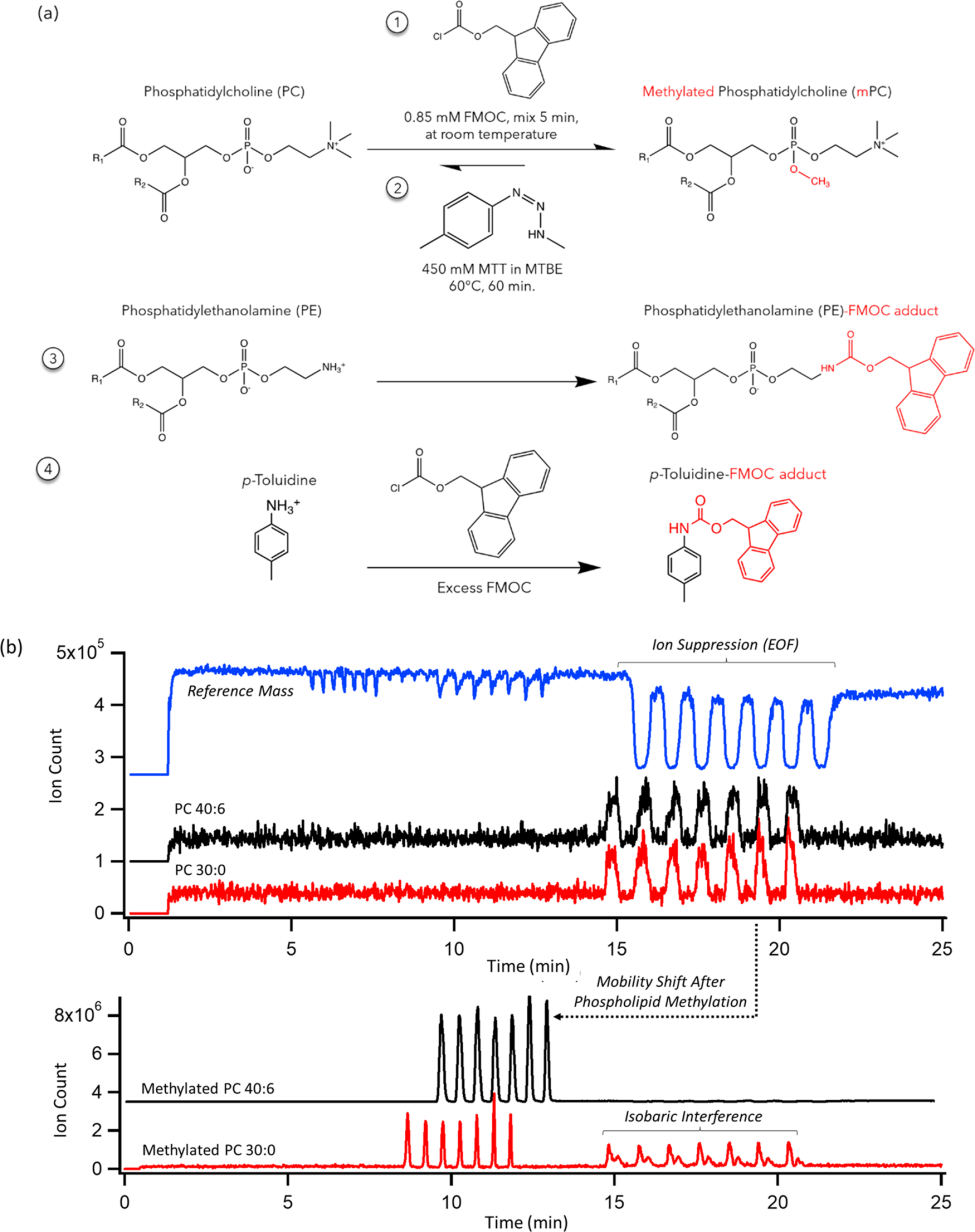
(a) Overview of the FMOC/MTT derivatization scheme proposed as a safer alternative to diazomethane to render zwitterionic PLs with a net positive charge as their methyl phosphate esters. The initial addition of excess FMOC reacts with interfering PEs to avoid isobaric interferences with PCs following methylation while also reacting with *p*-toluidine as a major byproduct in the reaction to reduce ion suppression prior to hexane back extraction. (b) Series of extracted ion electropherograms in MSI-NACE-MS under positive ion mode that highlight the large mobility shift following methylation, where methylated (cationic) phospholipids (PC) migrate faster than the EOF (blue) with improved resolution and separation efficiency. Major ion suppression for the reference mass is evident for native zwitterionic PCs comigrating close to the EOF, which is avoided after their chemical derivatization. Note that the PE-FMOC intermediate shown is subsequently methylated and converted into a neutral adduct after MTT addition.

A two-step chemical labeling strategy using FMOC/MTT was first developed to generate a positive charge on methylated PLs to increase their electrophoretic mobility, as depicted in Figure 1a. FMOC was first added as a protecting agent to rapidly react (<5 min) with phosphatidylethanolamines (PEs) from plasma ether extracts since they can generate isobaric interferences with analogous PCs following their permethylation.^44^ In this case, MSI-NACE-MS under alkaline buffer conditions and negative ion mode can directly analyze native PEs and other acidic lipids without chemical derivatization.^38^ FMOC reacts not only with PE species from plasma ether extracts but also with excess MTT byproducts (i.e., *p*-toluidine) to form a neutral adduct as shown in the proposed reaction mechanism (Figure S1). The reaction of *p*-toluidine with FMOC (Figure S2) contributes to a reduction of ion suppression for closely migrating methylated phospholipids in MSI-NACE-MS in conjunction with back extraction into hexane that was found to be superior to MTBE as an organic solvent (Figure S3). Overall, methylation of phosphoric acid moieties expands the separation window in MSI-NACE-MS by improving the resolution within PL class species, as shown in Figure 1b. Furthermore, cationic methylated PCs migrate with faster migration times and sharper peaks that enhance concentration sensitivity while avoiding ion suppression that occurs predominately within the EOF region due to the comigration of abundant and electrically neutral plasma lipids (*e.g*., diacylglycerides, cholesteryl esters, etc.). In all cases, a serial injection of seven independent plasma extracts was analyzed rapidly within a single analytical run by MSI-NACE-MS (∼3.5 min/sample) under positive ion mode with full-scan data acquisition. This method also analyzed methylated SM species, which also undergo a distinct mobility and mass shift (+14 Da) as shown in their MS/MS spectra acquired under positive and negative ion mode detection (Figure S4). SMs have been reported to undergo methylation with a second equivalent on their hydroxyl moiety when using diazomethane, which leads to signal splitting and lower sensitivity gain.^42^ In our case, dimethylated SM species were not detected likely due to the lower reactivity of MTT compared to diazomethane that requires special safety precautions when handling, given its explosive hazards and toxicity.^42−44^

### Optimization of FMOC/MTT Derivatization of Plasma Phospholipids

**Figure 2:**
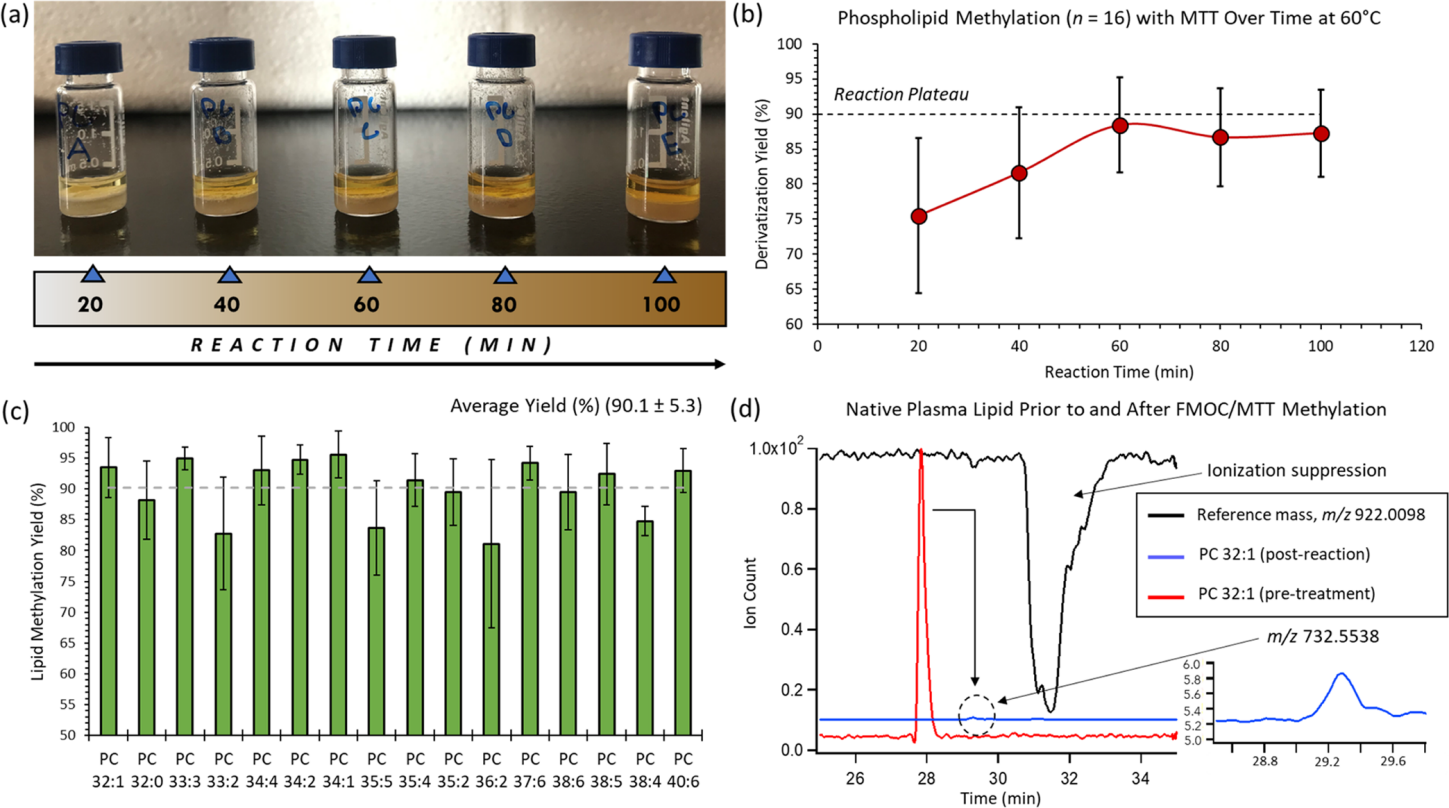
(a) Optimization of FMOC/MTT derivatization conditions as a function of the reaction time highlights a visible change in the yellow color intensity with longer reaction times. (b) A minimum reaction time of 60 min at 60 °C was determined to generate a quantitative and stable yield of methylated PCs based on analysis of 16 representative plasma PCs from NIST SRM-1950. (c) Bar graphs that compare the average yield of methylated PCs (∼90%) in plasma extracts, where errors bars represent standard deviation (±1 s, *n* = 5). (d) Representative extracted ion electropherograms highlighting the quantitative yield of methylated PCs without ion suppression. Reaction yields were assessed on native (underivatized) plasma PCs analyzed prior to and following FMOC/MTT labeling using NACE-MS with a single sample injection to improve their resolution from the EOF and avoid matrix-induced ion suppression effects.

MTT was previously introduced as a methylation agent for esterification of carboxylic acids^47^ that allowed for the analysis of acidic metabolites in urine by GC-MS.^48^ Similarly, Furukawa et al.^49^ reported using MTT to methylate oligosaccharides containing sialic acid residues in glycoblotting experiments prior to MALDI-MS analyses. However, this reagent remains unexplored to date, with sparse information related to its reaction mechanism and applicability to routine lipidomic analyses. Initial studies were performed to optimize reaction conditions for the formation of methylated PCs as a function of three experimental factors, namely, the reaction time (0–180 min), MTT concentration (50–900 mM), and reaction temperature (20–100 °C). A maximum yield for methylated PCs was achieved using 450 mM MTT with a reaction time of 60 min at 60 °C corresponding to an average yield of ∼70%. This apparent reaction yield was lower than first anticipated without the use of FMOC due to ion suppression effects from *p*-toluidine formed as a byproduct when using excess MTT (data not shown). A kinetic study was next performed to determine the minimum reaction time needed when using a two-step chemical derivatization strategy based on FMOC/MTT, where the reaction progress was reflected by a more intense golden/amber hue, as shown in Figure 2a. Also, Figure 2b highlights that the reaction yield plateaued at 60 min as shown for 16 representative plasma PCs species analyzed from NIST SRM-1950 when using MSI-NACE-MS. Importantly, the use of FMOC and hexane back extraction alleviated the issues of isobaric lipid interferences and ion suppression effects, resulting in higher and more consistent quantitative reaction yields (90.1 ± 6.4)% as demonstrated in Figure 2c. In some instances, the use of FMOC nearly doubled the reaction efficiency for certain methylated PCs (e.g., PC 36:5, PC 36:4, PC 40:6) as they only had an apparent ∼45% reaction yield when using MTT alone. The derivatization yield was assessed by taking the ratio of the normalized signal for each underivatized PC prior to and after FMOC/MTT treatment of NIST SRM-1950 human plasma (refer to eq 1) when using a conventional single sample injection format in NACE-MS. This process ensured that native PCs were adequately resolved from the EOF to avoid ion suppression, as highlighted for PC 32:1 in Figure 2d. However, a limitation of the hexane back extraction protocol following FMOC/MTT derivatization was that more polar lipid classes from plasma extracts were not adequately recovered, including shorter-chain PCs (<30:0) and lysophosphatidylcholine (lysoPCs). However, most of these polar PC species can be directly analyzed by MSI-NACE-MS under negative ion mode detection without derivatization.^38^ Similarly, plasma lipidomic protocols that rely on more polar organic solvent mixtures for single-phase extraction often suffer from limited recovery and poor solubility for nonpolar lipids that prevents their accurate quantification.^50^

### Expanded Lipidome Coverage and Phospholipid Classification via Mobility Maps

**Figure 3:**
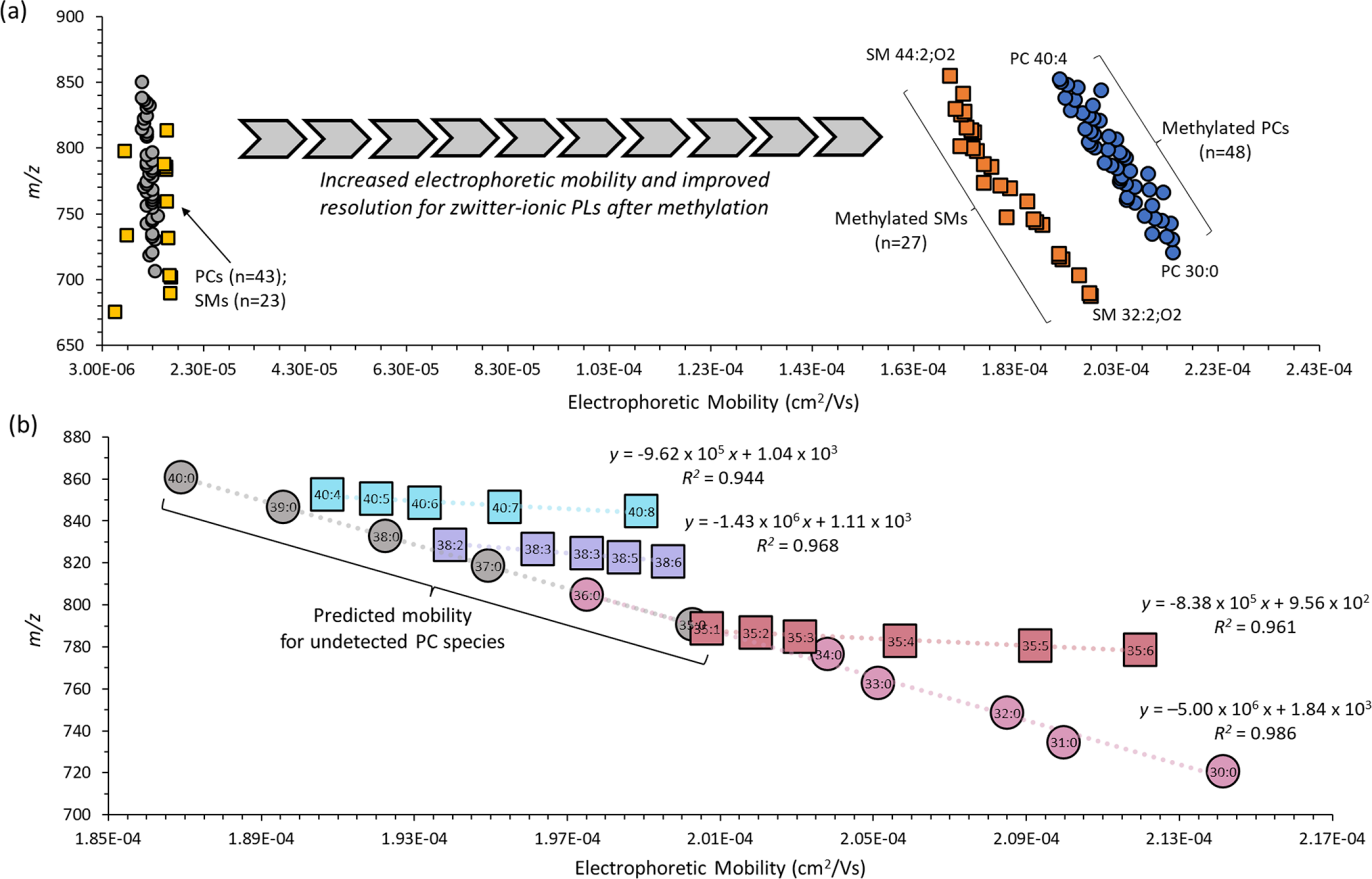
(a) Electrophoretic mobility plot as a function of the accurate mass for 76 PLs measured in NIST SRM-1950 plasma extracts by MSI-NACE-MS under positive ion mode detection. A large mobility shift occurs following quantitative methylation, resulting in better separation resolution of both methylated PCs and SMs that is dependent on their chemical linkage, total fatty acyl chain carbon numbers, and degrees of unsaturation. (b) Linear least-squares regression models were used to predict changes in the apparent electrophoretic mobility for plasma PLs as reflected by a homologous series of saturated and polyunsaturated PCs with the same total carbon chain length and as a function of increasing degrees of unsaturation. These distinctive mobility trends support the identification of unknown lipids in conjunction with MS/MS.

Similar to the use of collisional cross-sectional areas for classifying lipid structures as gas-phase ions in IMS,^31^ the electrophoretic mobility represents an intrinsic physicochemical parameter for characterizing ionic lipids in solution by MSI-NACE-MS.^38^ Zwitterionic PC species that migrate close to the EOF under alkaline BGE conditions overlap substantially, resulting in a narrow separation window as compared to acidic lipid classes, such as PEs, phosphatidylinositols (PIs), lysophosphatidic acids (LPAs), and free/nonesterified fatty acids (FAs). This scenario was suboptimal for PCs and SMs as it can contribute to false discoveries from isobaric interferences when performing untargeted lipidomics. Figure 3a highlights that a large mobility shift with improved separation resolution occurred following FMOC/MTT derivatization for two major classes of PLs, namely, methylated PCs (*n* = 48) and SMs (*n* = 27). These plasma PLs were annotated based on their sum composition, mass error (<10 ppm), and relative migration times (RMTs) or apparent electrophoretic mobilities (Tables S1 and S2).

Moreover, these cationic phospholipids also satisfied our selection criteria when using temporal signal pattern recognition in MSI-NACE-MS to reject spurious signals and background ions,^38^ which were also independently verified as consensus plasma lipids in an interlaboratory harmonization study using NIST SRM-1950.^15^ In general, methylated SMs migrated with a slower positive mobility than PCs due to differences in their chemical linkage bonding that impact their conformational size in solution. Among methylated PC and SM species having similar masses (i.e., PC 32:2 ≈ SM 36:2;O2), the SMs migrated later due to their longer acyl chains, resulting in a slower overall electrophoretic mobility in solution. Also, there were characteristic mobility shift patterns evident within both PL subclasses^38^ since a longer fatty acyl backbone (C30–C44) and greater degrees of unsaturation (*n* = 0–8) predictably reduce or increase the apparent mobility for methylated PCs and SMs, respectively, as previously shown for various acidic lipids and FAs.^36,38^ The separation resolution of native zwitterionic PLs under these conditions was otherwise poor in MSI-NACE-MS as they comigrate close with the EOF. The steepness of the slope for underivatized PLs reflects their inadequate within-class separation, which are also prone to ion suppression effects. The benefit of methylation of plasma PLs is more clearly illustrated in Figure 3b, which compares mobility changes among saturated PCs (including predicted mobility for nondetected PCs via extrapolation), as well as a homologous series of PC 36, PC 38, and PC 40 that demonstrate a linear increase in their positive electrophoretic mobility as a function of higher degrees of unsaturation when using a least-squares linear regression model (*R*^2^ > 0.930). Despite their similar charge state, more highly unsaturated methylated PCs in this case have smaller hydrodynamic sizes in solution than less unsaturated or fully saturated homologues.

**Figure 4:**
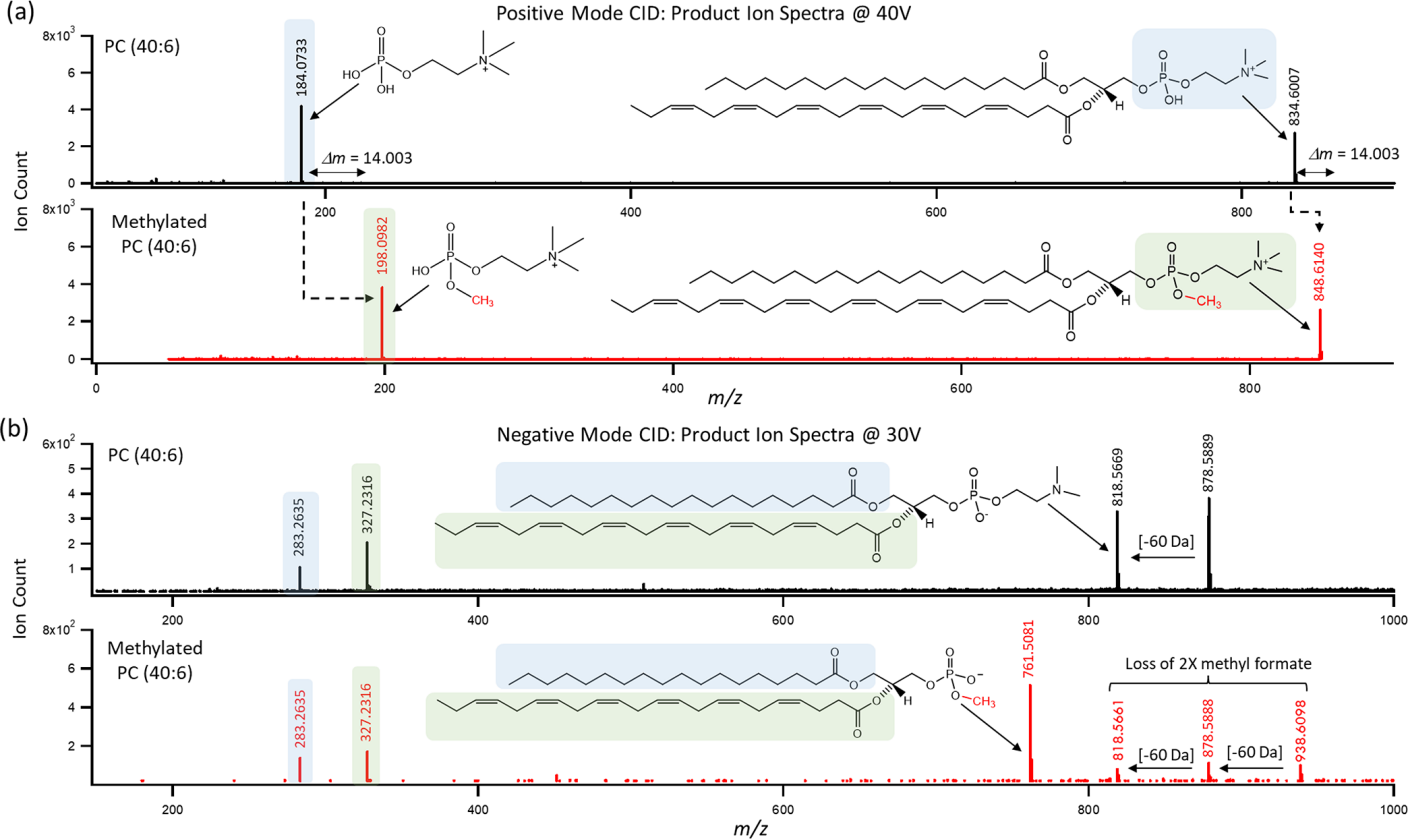
Comparison of MS/MS spectra acquired after collision-induced dissociation experiments under (a) positive and (b) negative ion modes for methylated and native PC 40:6 from plasma extracts. This confirmed the methylation of the phosphatidylcholine headgroup as reflected by a characteristic methyl shift (+14 Da) when comparing the molecular ion and base peak/product ion under positive ion mode, whereas the fatty acyl chain backbone and their relative positioning under negative ion mode was consistent with PC 18:0_22:6.

Figure 4 confirms that the large mobility shift was a result of formation of a cationic phosphate methyl ester, as shown in the MS/MS spectra acquired for PC 40:6 under positive and negative ion mode. Annotation of the MS/MS spectra under positive ion mode (at 40 V) for methylated PC 40:6 relative to native PC 40:6 confirmed a diagnostic product ion for its methylated phosphate headgroup (*m*/*z* 198.0982) corresponding to a mass shift of *m*/*z* 14 compared to the native PC (*m*/*z* 184.0773). Also, annotation of the MS/MS spectra acquired under negative ion mode (at 30 V) confirmed that both methylated PC 40:6 and native PC 40:6 contained stearic acid (18:0) and docosahexaenoic acid (22:6, DHA) with the latter likely from an sn-2 position when comparing the signal fragment ratio for the two fatty acyl chains. Interestingly, a double formate adduct anion [M + 2OOCH_3_]^−^ was detected as the molecular ion for methylated PC 40:6 (PC 18:0_22:6) when acquiring MS/MS spectra in negative ion mode since formic acid was included as an electrolyte in the BGE and sheath liquid solution. This was reflected by a characteristic neutral loss of *m*/*z* 60 (methylformate) that occurred twice compared with only once for native PC 40:6. Moreover, methylated PC 40:6 generated a unique base peak product ion at *m*/*z* 761.5081 in negative ion mode unlike native PC 40:6. However, not all methylated PC isomers from NIST SRM-1950 plasma extracts were comprised of fully resolved species in MSI-NACE-MS as highlighted for methylated PC 38:5 after acquiring MS/MS spectra under negative ion mode (Figure S5), which comprised a mixture of two comigrating PL species, namely, PC 16:0_22:5 and PC 18:1_20:4. Distinctive MS/MS spectra were also acquired for methylated SM 34:1;O2 under positive and negative ion mode conditions (Figure S4) that confirmed the same methylated phosphorylcholine headgroup but lacked diagnostic fatty acyl chains, which may be better achieved as their lithiated adducts to lower the energy barrier in collision-induced dissociation.^51^ Other approaches are needed to confirm the exact stereochemistry of methylated PL molecular species and their potential isomers from human plasma extracts, such as the location of unsaturation and/or geometric configuration when using MS/MS in conjunction with ozone-induced dissociation experiments^52^ or photochemical derivatization.^53^ Nevertheless, mobility plots generated separately for a series of methylated PCs and SMs provide complementary information to deduce the probable chemical structure of plasma PLs and reject potential isobaric candidates compared to relying on accurate mass alone (Figure S6). Overall, MSI-NACE-MS combines the selectivity of HILIC (i.e., polar headgroup/chemical linkage) and reversed-phase (*i.e*., total carbon chain length) chromatography, which is optimal for the rapid analysis of ionic classes of lipids from volume or mass-limited samples.^38^

### Characterization of Consensus Phospholipids from Reference Human Plasma

Previously, Bowden et al.^15^ reported the use of NIST SRM-1950 as a reference sample when comparing the performance of untargeted lipidomic platforms across 31 international laboratories, each using their own analysis data workflows, LC-MS methodology, and hardware/software configuration. Although 1527 unique lipid features were measured quantitatively across all sites, only 339 of these plasma lipids were reported consistently from at least five or more laboratories with adequate precision based on a minimum coefficient of dispersion threshold (COD < 40%). We next aimed to validate our two-stage chemical derivatization protocol using MSI-NACE-MS for a panel of methylated PCs and SMs measured consistently from NIST SRM-1950 plasma extracts compared to various standardized LC-MS protocols. Overall, 75 plasma PLs reported in the harmonization study were annotated based on their sum composition from NIST SRM-1950 ether extracts in a targeted manner, including 48 PCs and 27 SMs as their cationic phosphate methyl esters (Tables S1 and S2). Overall, MSI-NACE-MS was able to measure 90% of reported consensus PCs (48 out of 53) and SMs (27 out of 30) from NIST SRM-1950, respectively, based on the combined PL annotations used by Bowden et al.,^15^ which also included mass resolvable plasmanyl and plasmenyl species. However, the latter lipid species were confirmed not to be detected in our case. An analysis of acidic lipids from NIST SRM-1950 was also performed when using MSI-NACE-MS under negative ion mode without chemical derivatization to expand lipidome coverage to include more polar classes of acidic lipids under alkaline conditions.^38^ This also includes LPCs that have poor recovery after hexane back extraction and PEs that generate isobaric interferences with PCs after methylation if FMOC was not used as a protecting agent. In this case, we were able to reliably measure 11/14 (79%) bile acids (BAs) and 19/25 (76%) of LPCs but only 24/35 (69%) PE and 7/13 (54%) PI species from the consensus plasma lipids reported by Bowden et al.^15^ The reduced coverage was likely due to the lower ionization efficiency of polar/acidic lipids under negative ion mode detection in conjunction with the much smaller sample volumes introduced in-capillary (∼10 nL) in MSI-NACE-MS than LC-MS methods. Although only 8 FA species satisfied the selection criteria in the lipidomics harmonization study, MSI-NACE-MS can quantify more than 20 FAs from blood extracts as described elsewhere.^6,53^Figure S7 depicts a Venn diagram for consensus PLs from NIST SRM-1950 that were measured by MSI-NACE-MS under both positive and negative ion mode. As expected, a larger fraction (∼50%) of methylated PCs and SMs were measured consistently by MSI-NACE-MS in positive ion mode relative to negative ion mode without chemical derivatization. This was due to the improved separation resolution and greater ionization response achieved for cationic PCs and SMs following FMOC/MTT derivatization and hexane back extraction. Overall, our work highlights that >150 ionic lipids can be measured in reference plasma by MSI-NACE-MS under two complementary configurations, including phosphatidylserines (PSs) and PAs that were not reported as consensus plasma lipids when using LC-MS methods.^15^ For comparison, large-scale CE-MS metabolomic studies using aqueous BGE conditions typically measure <90 polar/hydrophilic metabolites consistently in blood specimens under positive and negative ion modes when using a coaxial sheath liquid flow interface.^39,54^

### Semiquantification of Phospholipids via Consensus Concentrations in Reference Plasma

**Figure 5:**
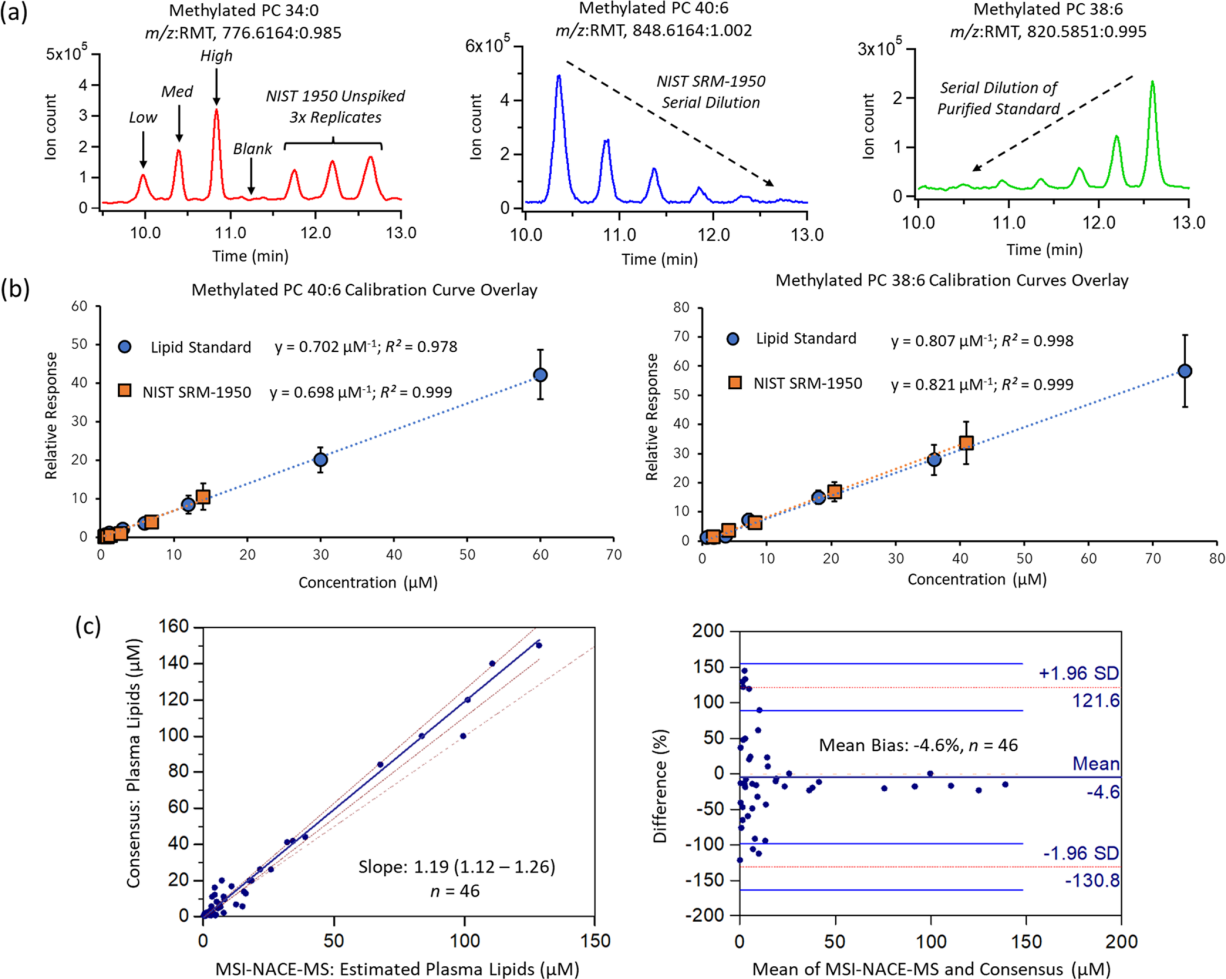
(a) Representative extracted ion electropherograms for methylated PC species when using distinct serial injection configurations in MSI-NACE-MS, including spike and recovery studies, serial dilution of NIST SRM-1950, and serial dilution of calibrant solutions. (b) The lack of ion suppression effects for methylated PC 40:6 and PC 38:6 was evident based on the good mutual agreement of their relative response factors or the slope (i.e., μM^–1^) acquired from 5-point calibration curves after serial dilution of PL standards or NIST SRM-1950. (c) Interlaboratory method comparison of PCs (*n* = 20) and SMs (*n* = 26) as consensus PLs from NIST SRM-1950 reported by Bowden et al. relative to their average concentrations measured by MSI-NACE-MS. Plasma PL concentrations were estimated by performing a serial dilution of NIST SRM-1950 using their median of mean concentrations (>0.5 μM, COV < 40%) to derive a response factor in MSI-NACE-MS among 21 quantifiable PLs (>4 calibrant points, Table 1). This strategy allowed for semiquantification of plasma PLs by MSI-NACE-MS when standards were lacking. However, greater variability and bias were noted for lower abundance plasma PLs with an average bias of 103% (<5 μM, *n* = 17), whereas an average bias of −9.7% was more acceptable for more abundant PLs (>5.0 μM, *n* = 29). As expected, plasma SMs and PLs using surrogate lipids for response factor estimation were more prone to inaccuracy.

A major analytical challenge in contemporary lipidomic research remains reliable quantification, given the lack and/or high costs of lipid standards and matching stable-isotope internal standards. However, a key advantage of MSI-NACE-MS is that ionic lipids migrate with a steady-state mobility under isocratic BGE conditions while using a continuous sheath liquid solution during ionization, unlike LC-MS methods that rely on gradient elution for optimal separation performance. Multiplexed separations in MSI-NACE-MS not only improve sample throughput but also enable versatile serial sample injection configuration to encode mass spectral information temporally within a separation,^38^ which reduces mass ambiguities when credentialing ionic lipids in an untargeted manner.^55^Figure 5a highlights that different serial injection configurations can be designed in MSI-NACE-MS within a single run, such as a spike recovery study for methylated PC 34:0 in NIST SRM-1950 human plasma, a serial dilution of NIST SRM-1950 to estimate the relative response ratio of methylated PC 40:6, and a serial dilution of a lipid standard for methylated PC 38:6 for generation of an external calibration curve. Spike and recovery experiments using four PC lipid standards were also performed at three different concentration levels (low, medium, and high) ranging from 1.0 to 20 μM (*n* = 5). In all cases, methylated PCs and SMs were normalized to a single deuterated internal standard, given the lack of ion suppression or enhancement effects in MSI-NACE-MS after sample workup. The potential for reliable quantification of methylated PCs was evaluated by comparing relative response factors (i.e., sensitivity) generated from the slopes of the calibration curves for each lipid standard with those derived for the same lipid following a serial dilution of NIST SRM-1950 human plasma. In the latter case, consensus (median of mean) PL concentrations reported in a lipidomics harmonization study^15^ were used to construct calibration curves. Figure 5b depicts two representative calibration curve overlays for methylated PC 38:6 and PC 40:6, which highlights good mutual agreement in measured sensitivity (i.e., slope of the calibration curve) based on a least-squares linear regression with excellent linearity (*R*^2^ > 0.980). This comparison also confirmed the lack of matrix-induced ion suppression in MSI-NACE-MS, given minimal differences (bias <2%) in the apparent sensitivity measured from calibrant standards relative to reference plasma extracts.

Table S3 summarizes the performance of MSI-NACE-MS for reliable quantification of four representative plasma PCs when external calibration curves were used compared to a serial dilution of NIST SRM-1950. As expected, good accuracy was achieved when quantifying methylated PC 34:0, PC 38:6, and PC 40:6 in both spike recovery studies as well as unspiked reference plasma (mean bias <10%) when using calibration curves by MSI-NACE-MS when compared to untargeted LC-MS methods.^15^ A slightly higher bias (<25%) was found for PC 38:6 and PC 40:6 concentrations in NIST SRM-1950 when compared to a targeted shotgun lipidomic interlaboratory comparison study by DI-MS/MS using a commercial lipid kit under standardized operating conditions.^17^ The latter discrepancy may arise due to isobaric interference when high-efficiency separations are not used in lipidomic analyses. Overall, poor accuracy (mean bias ∼−50%) was noted primarily for PC 30:0 after hexane sample cleanup since this procedure favors a quantitative recovery of more lipophilic PLs having longer total carbon acyl chain lengths.

We also explored an alternative strategy for semiquantitative estimation of other plasma PLs lacking chemical standards via response factors derived from the serial dilution of NIST SRM-1950 when using the median of mean consensus lipid concentrations reported by Bowden et al.^15^ As expected, this strategy was better suited to more abundant plasma PLs (>10 μM), given the serial dilution process unlike lipid standards that permitted PL quantification over a wider linear dynamic range (Figure 5b). Overall, 21 plasma PC (*n* = 14) and SM (*n* = 7) species were measured in at least 4 concentration levels with adequate precision (CV < 20%) and linearity (mean *R*^2^ = 0.987) as summarized in Table 1. This in turn was used to estimate the response factors and corresponding concentrations for 46 annotated plasma PLs (>0.5 μM), including 19 PCs and 27 SMs (Table S4). In cases where a direct measurement of a response factor was not feasible by MSI-NACE-MS due to inadequate dynamic range, the closest PL analogue in terms of mass and lipid class from Table 1 was used as a surrogate to estimate its response factor. Figure 5c demonstrates that this approach generally resulted in a good mutual agreement when estimating the concentration for most plasma PLs by MSI-NACE-MS compared to their consensus concentrations by several LC-MS methods as reflected by a slope of 1.19 (95% CI: 1.12–1.26) and a mean bias of −6.9% over a 500-fold dynamic range (0.5–200 μM). Yet, greater bias and variability were evident for lower abundance PLs (<5 μM) as response factors were more difficult to reliably assess in MSI-NACE-MS following serial dilution of NIST SRM-1950, resulting in the reliance of nonmatching PL surrogate species. For instance, the average bias was acceptable at −9.7% for most plasma PLs (*n* = 27) having reported consensus concentrations >5.0 μM in contrast to a larger average bias of 104% for PLs < 5.0 μM (*n* = 17). The latter group of PLs comprised mostly lower abundance SMs and PCs that relied on surrogate PLs to estimate their response factor with greater uncertainty (Table S4). Further work is needed to evaluate the quantitative accuracy and long-term analytical performance of MSI-NACE-MS for plasma PLs when using FMOC/MTT derivatization. Nevertheless, this approach offers a higher-throughput approach for quantitative lipidomic analyses even in cases when standards are not available, which was recently applied to identify two specific circulating PCs as surrogate biomarkers of the omega-3 index following high-dose fish oil, docosahexaenoic acid, or eicosapentaenoic acid supplementation.^55^

**Table 1:** Plasma Phospholipids (PCs, *n* = 14; SMs, *n* = 7) from NIST SRM-1950 Measured by MSI-NACE-MS Following a Serial Dilution to Estimate Their Relative Response Factor Using Consensus Concentrations^15^

lipid species^a^	methylated *m/z*	consensus concentration^b^ (μM)	no. of laboratories detected	response factor^c^ (μM^–1^)	no. of calibrant data points^c^	linearity (*R*^2^)
PC 30:0	720.5538	1.6	11	0.618	3	0.999
PC 34:1	774.6008	120	19	1.451	6	0.989
PC 34:0	776.6164	2.1	12	1.438	4	0.979
PC 36:4	796.5850	150	19	0.862	6	0.993
PC 36:3	798.6008	100	17	1.176	6	0.992
PC 36:2	800.6164	140	18	1.495	6	0.990
PC 36:1	802.6320	26	17	2.17	5	0.990
PC 38:6	820.5850	41	18	0.821	5	0.999
PC 38:5	822.6008	42	18	0.896	5	0.990
PC 38:4	824.6164	84	18	1.093	5	0.990
PC 38:3	826.6320	26	14	2.01	5	0.981
PC 40:6	848.6164	14	17	0.702	4	0.978
PC 40:5	850.6320	6.7	18	1.695	5	0.989
PC 40:4	852.6476	2.9	18	1.961	5	0.993
SM 34:1;O2	717.5904	100	21	0.241	5	0.984
SM 36:1;O2	745.6218	20	22	0.320	4	0.992
SM 40:2;O2	799.6688	12	15	0.714	4	0.975
SM 40:1;O2	801.6844	20	17	0.74	4	0.983
SM 42:3;O2	825.6844	17	12	0.625	4	0.971
SM 42:2;O2	827.7000	44	18	0.579	5	0.970
SM 42:1;O2	829.7156	20	21	0.720	4	0.982

a Annotated lipid species/isobars from NIST SRM-1950 consistently measured by various LC-MS methods in an interlaboratory lipidomics harmonization study by Bowden et al.^15^

b Reported consensus plasma phospholipid concentrations determined by a median of the means from at least five different laboratories having an overall COV < 40%.

c Relative response factors for each plasma phospholipid species following a serial dilution of NIST SRM-1950 to derive a linear calibration curve by MSI-NACE-MS with a minimum of 4 concentration levels (except for PC 30:0).

In summary, expanded lipidome coverage was achieved in MSI-NACE-MS when using a two-step precolumn chemical derivatization strategy to convert zwitterionic PLs into their corresponding cationic methyl phosphate esters. This labeling procedure is quantitative and more convenient to use than diazomethane for PL methylation, which results in improved separation performance and ionization efficiency. Overall, 75 cationic PCs and SMs were characterized from reference human plasma with adequate precisionby MSI-NACE-MS following FMOC/MTT derivatization and hexane back extraction when compared to an international lipidomic harmonization study. Additionally, more than 69 other acidic and polar PLs from NIST SRM-1950 plasma extracts can also be measured by MSI-NACE-MS under negative ion mode without chemical derivatization. Additionally, polar lipid classes poorly retained in reversed-phase LC-MS (e.g., PAs, PSs, FAs) are well resolved in MSI-NACE-MS. This strategy greatly expands conventional CE-MS metabolomic protocols that rely on aqueous buffer systems and thus have been limited to the analysis of hydrophilic/polar metabolites. Lipid annotation and structural classification were also supported based on predictable trends in the electrophoretic mobility for methylated PCs and SMs that are dependent on polar headgroup/chemical linkage, total fatty acyl chain length, and degrees of unsaturation. Advantages of MSI-NACE-MS include greater throughput and minimal ion suppression effects that allow for unique data workflows for data acquisition and lipid authentication in comparison to other separation methods that utilize single sample injections. MSI-NACE-MS is also more amenable to standardization since it operates using only a bare fused-silica capillary under an isocratic nonaqueous buffer system unlike LC-MS that relies on different column types and gradient elution programs when using reversed-phase and HILIC separations. However, MSI-NACE-MS with a coaxial sheath liquid interface suffers from higher detection limits and lower concentration sensitivity for ionic lipids compared to LC-MS protocols due to the smaller sample volume introduced on-capillary. Also, electrically neutral lipid classes are not resolved or reliably measured even after methylation, such as diacylglycerides and cholesteryl esters. Future studies are underway to better characterize other methylated PL subclasses in MSI-NACE-MS with improved sensitivity while developing accelerated data workflows and automated data processing tools for biomarker discovery in lipidomic studies.

## Conclusions

In this work, we introduce a two-step chemical derivatization strategy using FMOC/MTT for the methylation of zwitterionic PLs to expand lipid profiling coverage by MSI-NACE-MS under positive ion mode conditions. FMOC was used as a compatible protecting agent to prevent generation of PE isobaric species to PCs that also reduced ion suppression effects from excess MTT byproducts prior to hexane back extraction. We optimized the efficacy of this reaction to generate quantitative yields of 75 cationic methylated PCs and SMs verified in reference human plasma when using MSI-NACE-MS, which comprised 90% of consensus plasma lipids within these two classes, as reported in an international lipidomics harmonization study. Overall, PL methylation resulted in improved separation resolution, faster analysis times, and reduced ion suppression while allowing for better lipid structural classification based on changes in their electrophoretic mobility. This method is optimal for lipidomic studies requiring higher sample throughput and lower operating costs with stringent quality control while consuming minimal volumes of the sample and organic solvent. Complementary analysis of other polar or acidic lipid classes can be achieved by their direct analysis using MSI-NACE-MS under negative ion mode without chemical derivatization. We also demonstrated good precision and accuracy when quantifying methylated PCs and SMs in reference plasma samples, including the potential for use of serial dilution of NIST SRM-1950 to estimate relative response factors for lipids lacking chemical standards, provided they are present at concentrations >5 μM. This methylation strategy is anticipated to offer a practical alternative to diazomethane for improved lipid analysis when using other MS instrumental platforms without excessive hazards and safety precautions, including direct infusion-MS, ion mobility-MS, and LC-MS/MS methods.

## Supporting Information Available

Additional reaction mechanisms, mass spectra, mobility plots, Venn diagram of lipidome coverage, and annotated methylated phospholipids and their estimated concentrations in reference plasma extracts using MSI-NACE-MS with FMOC/MTT labeling (PDF)

## References

[ref1] Quehenberger O.; Dennis E. A. N. Engl. J. Med. 2011, 365 (19), 1812–1823. 10.1056/NEJMra1104901. 22070478 PMC3412394

[ref2] Shevchenko A.; Simons K. Nat. Rev. Mol. Cell Biol. 2010, 11 (8), 593–598. 10.1038/nrm2934. 20606693

[ref3] Meikle T. G.; Huynh K.; Giles C.; Meikle P. J. J. Lipid Res. 2021, 62, 100127 10.1016/j.jlr.2021.100127. 34582882 PMC8528718

[ref4] Han X. Nat. Rev. Endocrinol. 2016, 12 (11), 668–679. 10.1038/nrendo.2016.98. 27469345

[ref5] Hyötyläinen T.; Bondia-Pons I.; Orešič M. Mol. Nutr. Food Res. 2013, 57 (8), 1306–1318. 10.1002/mnfr.201200759. 23413227

[ref6] Azab S. M.; de Souza R. J.; Teo K. K.; Anand S. S.; Williams N. C.; Holzschuher J.; McGlory C.; Philips S. M.; Britz-McKibbin P. J. Lipid Res. 2020, 61 (6), 933–944. 10.1194/jlr.D120000630. 32234835 PMC7269757

[ref7] Han X.; Gross R. W. J. Lipid Res. 2003, 44 (6), 1071–1079. 10.1194/jlr.R300004-JLR200. 12671038

[ref8] Kohno S.; Keenan A. L.; Ntambi J. M.; Miyazaki M. Biochem. Biophys. Res. Commun. 2018, 504 (3), 590–595. 10.1016/j.bbrc.2018.04.106. 29665359 PMC6309221

[ref9] Rustam Y. H.; Reid G. E. Anal. Chem. 2018, 90 (1), 374–397. 10.1021/acs.analchem.7b04836. 29166560

[ref10] McDonald J. G.; et al. Nat. Metab. 2022, 4 (9), 1086–1088. 10.1038/s42255-022-00628-3. 35934691

[ref11] O’Donnell V. B.; FitzGerald G. A.; Murphy R. C.; Liebisch G.; Dennis E. A.; Quehenberger O.; Subramaniam S.; Wakelam M. J. O. Circ.: Genomic Precis. Med. 2020, 13 (6), e003019 10.1161/CIRCGEN.120.003019. PMC8376269 33196315

[ref12] Drotleff B.; Lämmerhofer M. Anal. Chem. 2019, 91 (15), 9836–9843. 10.1021/acs.analchem.9b01505. 31241926

[ref13] Tsugawa H.; Ikeda K.; Takahashi M.; Satoh A.; Mori Y.; Uchino H.; Okahashi N.; Yamada Y.; Tada I.; Bonini P.; Higashi Y.; Okazaki Y.; Zhou Z.; Zhu Z. J.; Koelmel J.; Cajka T.; Fiehn O.; Saito K.; Arita M.; Arita M. A. Nat. Biotechnol. 2020, 38 (10), 1159–1163. 10.1038/s41587-020-0531-2. 32541957

[ref14] Delabriere A.; Warmer P.; Brennsteiner V.; Zamboni N. Anal. Chem. 2021, 93 (45), 15024–15032. 10.1021/acs.analchem.1c02687. 34735114

[ref15] Bowden J. A.; et al. J. Lipid Res. 2017, 58 (12), 2275–2288. 10.1194/jlr.M079012. 28986437 PMC5711491

[ref16] Barupal D. K.; Fan S.; Wancewicz B.; Cajka T.; Sa M.; Showalter M. R.; Baillie R.; Tenenbaum J. D.; Louie G.; Kaddurah-Daouk R.; Fiehn O. Sci. Data 2018, 5 (1), 180263 10.1038/sdata.2018.263. 30457571 PMC6244184

[ref17] Thompson J. W.; et al. Anal. Chem. 2019, 91 (22), 14407–14416. 10.1021/acs.analchem.9b02908. 31638379 PMC7310668

[ref18] Züllig T.; Trötzmüller M.; Köfeler H. C. Anal. Bioanal. Chem. 2020, 412 (10), 2191–2209. 10.1007/s00216-019-02241-y. 31820027 PMC7118050

[ref19] Abel K.; de Schmertzing H.; Peterson J. I. J. Bacteriol. 1963, 85 (5), 1039–1044. 10.1128/jb.85.5.1039-1044.1963. 14043992 PMC278281

[ref20] Han X.; Gross R. W. J. Lipid Res. 2022, 63 (2), 100164 10.1016/j.jlr.2021.100164. 34953866 PMC8953652

[ref21] Han X.; Gross R. W. Mass Spectrom. Rev. 2005, 24 (3), 367–412. 10.1002/mas.20023. 15389848

[ref22] Xu T.; Hu C.; Xuan Q.; Xu G. Anal. Chim. Acta 2020, 1137, 156–169. 10.1016/j.aca.2020.09.060. 33153599 PMC7525665

[ref23] Cajka T.; Fiehn O. Methods Mol. Biol. 2017, 1609, 149–170. 10.1007/978-1-4939-6996-8_14. 28660581

[ref24] Lange M.; Ni Z.; Criscuolo A.; Fedorova M. Chromatographia 2019, 82 (1), 77–100. 10.1007/s10337-018-3656-4.

[ref25] Harrieder E.-M.; Kretschmer F.; Böcker S.; Witting M. J. Chromatogr. B 2022, 1188, 123069 10.1016/j.jchromb.2021.123069. 34879285

[ref26] Narváez-Rivas M.; Zhang Q. J. Chromatogr. A 2016, 1440, 123–134. 10.1016/j.chroma.2016.02.054. 26928874 PMC4792668

[ref27] Plumb R. S.; Isaac G.; Rainville P. D.; Hill J.; Gethings L. A.; Johnson K. A.; Lauterbach J.; Wilson I. D. J. Proteome Res. 2022, 21 (3), 691–701. 10.1021/acs.jproteome.1c00836. 34968064

[ref28] Sorensen M. J.; Miller K. E.; Jorgenson J. W.; Kennedy R. T. J. Chromatogr. A 2020, 1611, 460575 10.1016/j.chroma.2019.460575. 31607445 PMC6980658

[ref29] Baglai A.; Gargano A. F. G.; Jordens J.; Mengerink Y.; Honing M.; van der Wal S.; Schoenmakers P. J. J. Chromatogr. A 2017, 1530, 90–103. 10.1016/j.chroma.2017.11.014. 29146423

[ref30] Wolrab D.; Chocholoušková M.; Jirásko R.; Peterka O.; Holčapek M. Anal. Bioanal. Chem. 2020, 412 (10), 2375–2388. 10.1007/s00216-020-02473-3. 32078000

[ref31] Leaptrot K. L.; May J. C.; Dodds J. N.; McLean J. A. Nat. Commun. 2019, 10 (1), 985 10.1038/s41467-019-08897-5. 30816114 PMC6395675

[ref32] Lee J.-H.; Kim S.-J.; Lee S.; Rhee J.-K.; Lee S. Y.; Na Y.-C. Anal. Chim. Acta 2017, 984, 223–231. 10.1016/j.aca.2017.06.052. 28843567

[ref33] Sándor V.; Berkics B. V.; Kilár A.; Kocsis B.; Kilár F.; Dörnyei Á. Electrophoresis 2020, 41 (13–14), 1178–1188. 10.1002/elps.201900251. 32335940

[ref34] Gao F.; Zhang Z.; Fu X.; Li W.; Wang T.; Liu H. Electrophoresis 2007, 28 (9), 1418–1425. 10.1002/elps.200600533. 17372939

[ref35] Montealegre C.; Sánchez-Hernández L.; Crego A. L.; Marina M. L. J. Agric. Food Chem. 2013, 61 (8), 1823–1832. 10.1021/jf304357e. 23379923

[ref36] Azab S.; Ly R.; Britz-McKibbin P. Anal. Chem. 2019, 91 (3), 2329–2336. 10.1021/acs.analchem.8b05054. 30570251

[ref37] Azab S. M.; de Souza R. J.; Ly R.; Teo K. K.; Atkinson S. A.; Morrison K. M.; Anand S. S.; Britz-McKibbin P. Prostaglandins, Leukotrienes Essent. Fatty Acids 2022, 176, 102378 10.1016/j.plefa.2021.102378. 34871861

[ref38] Ly R.; Ly N.; Sasaki K.; Suzuki M.; Kami K.; Ohashi Y.; Britz-McKibbin P. J. Proteome Res. 2022, 21 (3), 768–777. 10.1021/acs.jproteome.1c00682. 34676758

[ref39] Shanmuganathan M.; Kroezen Z.; Gill B.; Azab S.; Souza R. J. de.; Teo K. K.; Atkinson S.; Subbarao P.; Desai D.; Anand S. S.; Britz-Mckibbin P. Nat. Protoc. 2021, 16 (4), 1966–1994. 10.1038/s41596-020-00475-0. 33674789

[ref40] DiBattista A.; McIntosh N.; Lamoureux M.; Al-Dirbashi O. Y.; Chakraborty P.; Britz-McKibbin P. Anal. Chem. 2017, 89 (15), 8112–8121. 10.1021/acs.analchem.7b01727. 28648083

[ref41] Xia F.; Wan J.-B. Mass Spectrom. Rev. 2023, 42, 432–452. 10.1002/mas.21729. 34486155

[ref42] Wasslen K. V.; Canez C. R.; Lee H.; Manthorpe J. M.; Smith J. C. Anal. Chem. 2014, 86 (19), 9523–9532. 10.1021/ac501588y. 25208053

[ref43] Wasslen K. V.; Tan L. H.; Manthorpe J. M.; Smith J. C. Anal. Chem. 2014, 86 (7), 3291–3299. 10.1021/ac403349c. 24555738

[ref44] Betancourt S. K.; Canez C. R.; Shields S. W. J.; Manthorpe J. M.; Smith J. C.; McLuckey S. A. Anal. Chem. 2017, 89 (17), 9452–9458. 10.1021/acs.analchem.7b02271. 28764333 PMC5648332

[ref45] Dallinger D.; Kappe C. O. Nat. Protoc. 2017, 12 (10), 2138–2147. 10.1038/nprot.2017.046. 28906494

[ref46] Yamamoto M.; Ly R.; Gill B.; Zhu Y.; Moran-Mirabal J.; Britz-McKibbin P. Anal. Chem. 2016, 88 (21), 10710–10719. 10.1021/acs.analchem.6b03269. 27677202

[ref47] White E. H.; Baum A. A.; Eitel D. E. Org. Synth. 2003, 48, 102–105. 10.1002/0471264180.os048.30.

[ref48] Caperos J. R.; Fernández J. G. Occup. Environ. Med. 1977, 34 (3), 229–233. 10.1136/oem.34.3.229. PMC1008235 911693

[ref49] Furukawa T.; Hinou H.; Takeda S.; Chiba H.; Nishimura S.; Hui S. ChemBioChem 2017, 18 (19), 1903–1909. 10.1002/cbic.201700384. 28779513

[ref50] Höring M.; Stieglmeier C.; Schnabel K.; Hallmark T.; Ekroos K.; Burkhardt R.; Liebisch G. Anal. Chem. 2022, 94 (36), 12292–12296. 10.1021/acs.analchem.2c02117. 36048752 PMC9475500

[ref51] Hsu F.-F.; Turk J. J. Am. Soc. Mass Spectrom. 2000, 11 (5), 437–449. 10.1016/S1044-0305(99)00150-6. 10790848

[ref52] Claes B. S. R.; Bowman A. P.; Poad B. L. J.; Young R. S. E.; Heeren R. M. A.; Blanksby S. J.; Ellis S. R. Anal. Chem. 2021, 93 (28), 9826–9834. 10.1021/acs.analchem.1c01377. 34228922 PMC8295983

[ref53] Ma X.; Zhang W.; Li Z.; Xia Y.; Ouyang Z. Acc. Chem. Res. 2021, 54 (20), 3873–3882. 10.1021/acs.accounts.1c00419. 34570464

[ref54] Harada S.; Hirayama A.; Chan Q.; Kurihara A.; Fukai K.; Iida M.; Kato S.; Sugiyama D.; Kuwabara K.; Takeuchi A.; Akiyama M.; Okamura T.; Ebbels T. M. D.; Elliott P.; Tomita M.; Sato A.; Suzuki C.; Sugimoto M.; Soga T.; Takebayashi T. PLoS One 2018, 13 (1), e0191230 10.1371/journal.pone.0191230. 29346414 PMC5773198

[ref55] Ly R.; MacIntyre B. C.; Philips S. M.; McGlory C.; Mutch D. M.; Britz-McKibbin P. J. Lipid Res. 2023, 64 (11), 100445 10.1016/j.jlr.2023.100445. 37730162 PMC10622695

