# Supplementary material for: Expanding Lipidomic Coverage in Multisegment Injection–Nonaqueous Capillary Electrophoresis–Mass Spectrometry via a Convenient and Quantitative Methylation Strategy
Source: Anal Chem. 2023 Nov 22;95(48):17513–24. doi: 10.1021/acs.analchem.3c02605 (PMC10701711; doi:10.1021/acs.analchem.3c02605)
Supplement: Supplementary file 1 — ac3c02605_si_001.pdf [file ac3c02605_si_001.pdf]

# Supporting Information

## Expanding Lipidomic Coverage in Multisegment Injection-Nonaqueous Capillary Electrophoresis-Mass Spectrometry via a Convenient and Quantitative Methylation Strategy

*Ritchie Ly, Lucas Christian Torres, Nicholas Ly, Philip Britz-McKibbin**

*Department of Chemistry and Chemical Biology, McMaster University, Hamilton, Canada L8S 4M1*

*Corresponding author, Email: britz@mcmaster.ca

**Supplemental Figures S1-S7**

**Supplemental Tables S1-S4**

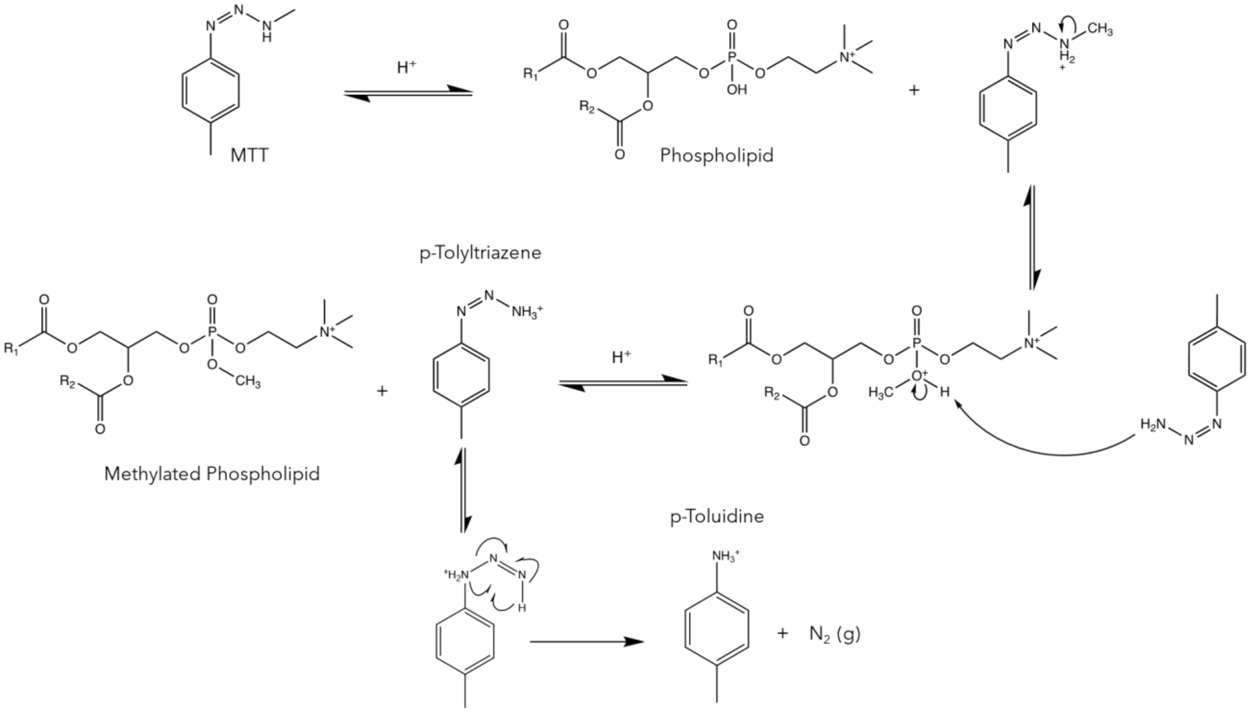


**Figure S1.** Proposed mechanism for methylation of phospholipids using MTT. Briefly, this reaction involves a proton transfer to amino group of MTT for activation to increase its electrophilic character resulting in formation of *p*-tolyltriazene and a methylated phosphate ester head group with a net cationic charge on the phospholipid. This reaction subsequently liberates $N_2$ gas with concomitant generation of *p*-toluidine as a major by-product.

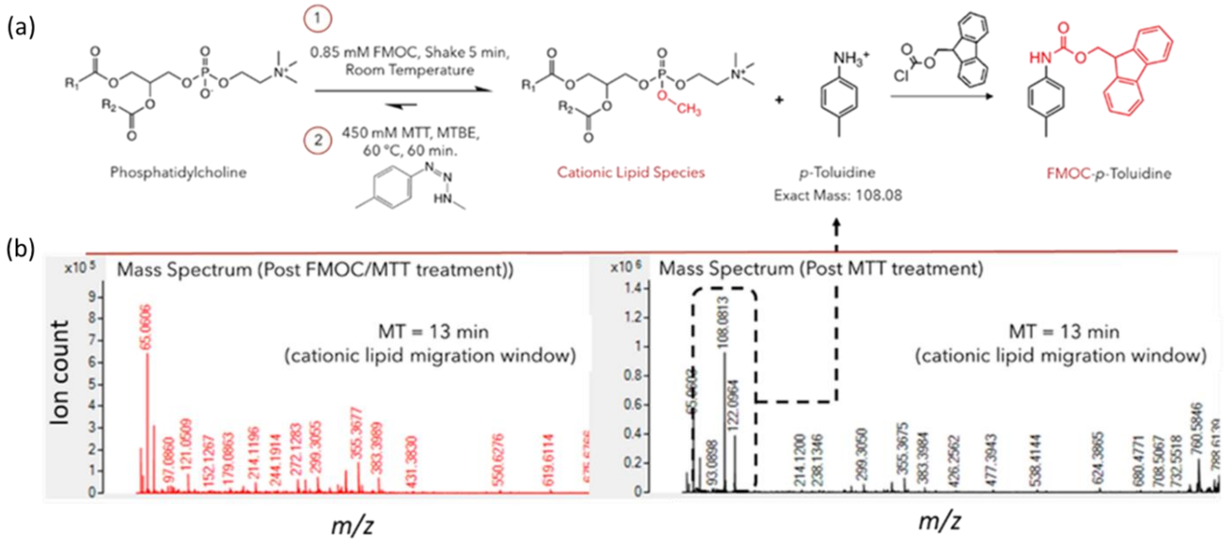


**Figure S2. (a)** Reaction scheme depicting *p*-toluidine by-product generation after methylation of phospholipids from MTT. **(b)** Prior to the introduction of FMOC, a singly charged molecular ion $[M+H]^+$ associated with formation of *p*-tolidine (*m/z* 108.081) was observed within the migration time (MT) separation window for methylated PC species. FMOC not only was required to react with PEs to prevent interference with isobaric PC species, but also to react with *p*-tolidine and form a neutral adduct, thereby preventing ion suppression as excess *p*-toluidine otherwise migrated close to methylated phospholipids in MSI-NACE-MS.

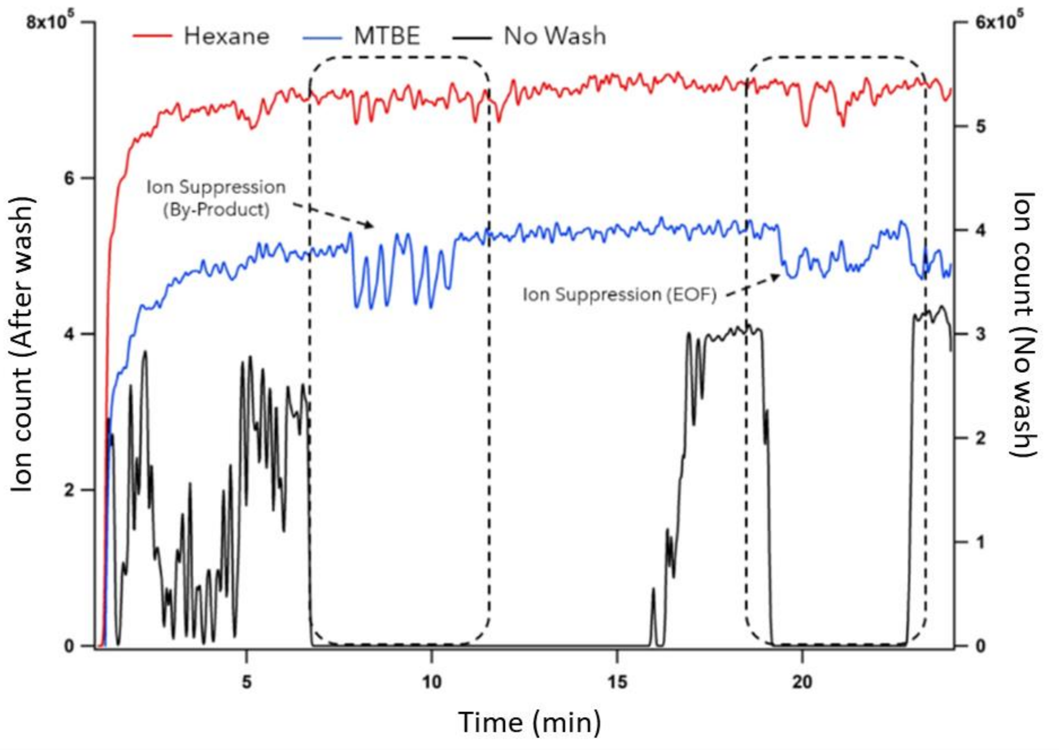


**Figure S3.** An extracted ion electropherogram overlay of purine (*m/z* 121.0509) used as reference mass calibrant in the sheath liquid solution under different sample workup conditions following FMOC/MTT derivatization of NIST SRM-1950 plasma extracts by MSI-NACE-MS under positive ion mode detection. Two major regions of ion suppression correspond to excess MTT by-product (e.g., *p*-toluidine) and the EOF, where abundant neutral lipid classes co-migrate (*e.g.*, cholesterol esters, diacylglycerides). Two different organic solvents (*i.e.*, MTBE vs. hexane) were used for back extraction of methylated phospholipids following chemical derivatization in order to reduce ion suppression effects as compared to a standard run without washing (black trace). Notably, the use of hexane (red trace) outperformed MTBE (blue trace) resulting in a superior sample cleanup that greatly reduced ion suppression from excess reagents in a region where methylated phospholipids migrate when using multiplexed separations by MSI-NACE-MS.

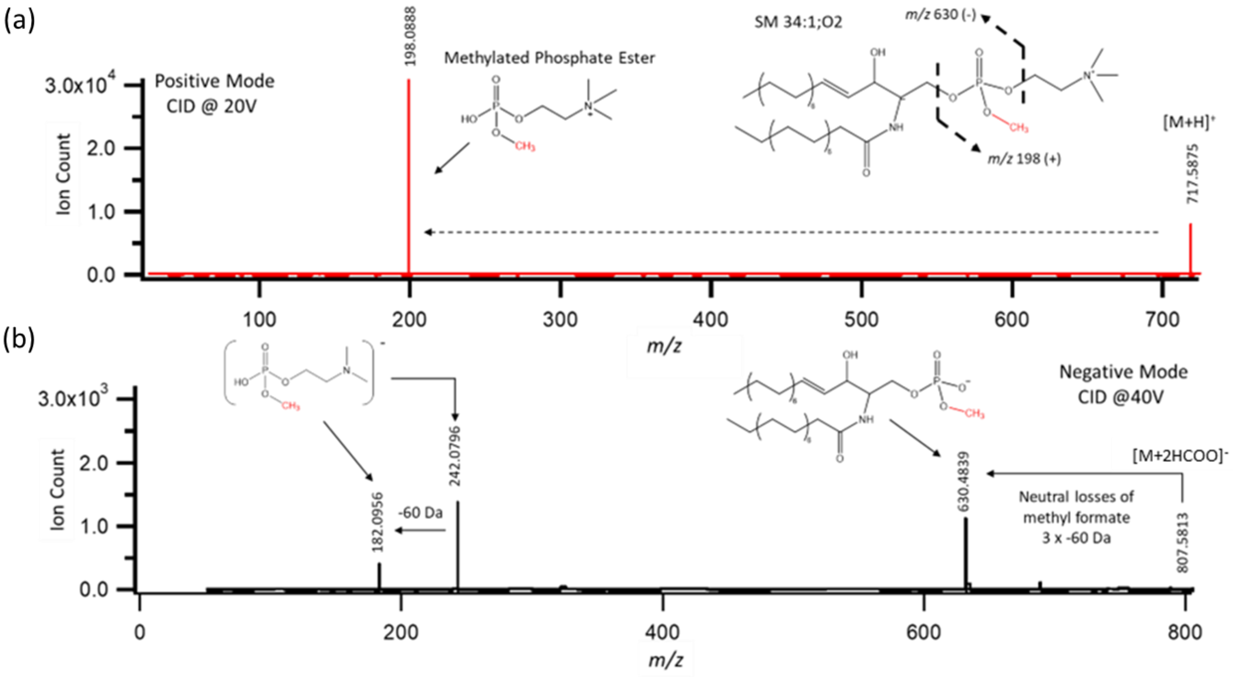


**Figure S4.** MS/MS spectra acquired after CID experiments on derivatized methylated SM 34:1;O2 in positive and negative ion mode when using NACE-MS (single injection). **(a)** For positive ion mode, a methyl shift [+14 Da] as shown in the base peak product ion (*m/z* 198.0888). **(b)** A double formate adduct anion for methylated SM 34:1;O2 were generated in negative ion mode, however acyl fatty acid product anions were not detected at this collision energy unlike methylated PCs.

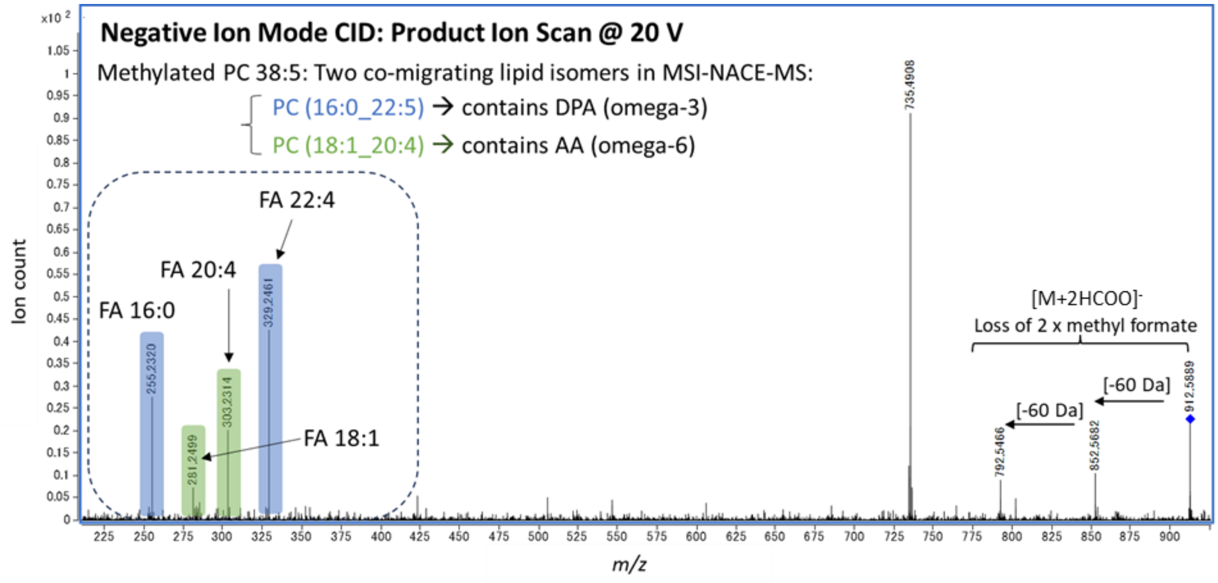


**Figure S5.** A representative MS/MS spectrum acquired for methylated PC 38:5 from SRM-1950 plasma ether extract when using MSI-NACE-MS, which was tentatively identified as an EPA (20:5)-containing phospholipid. However, collision-induced dissociation experiments at 20 V under negative ion mode subsequently confirmed that methylated PC 38:5 was in fact comprised of a mixture of two co-migrating phospholipids, namely PC (16:0_22:5) and PC (18:1_20:4) as major and minor species, respectively. As a result, not all plasma phospholipids annotated based on their sum composition represent fully resolved and unique lipid species.

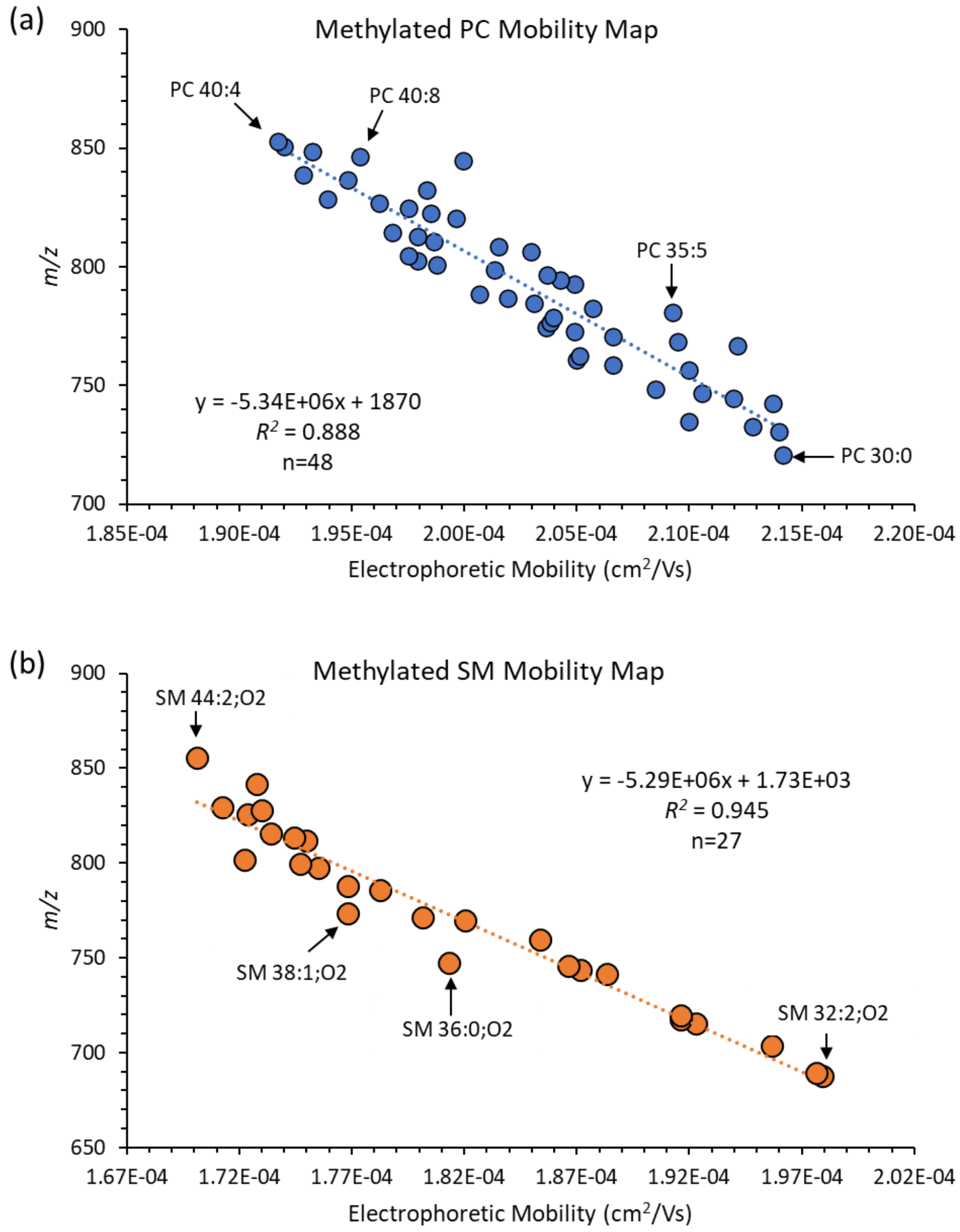


**Figure S6. (a)** Mobility map depicting all methylated PC species (n=48) and **(b)** methylated SM species (n=27) detected from NIST SRM-1950 human plasma extracts after FMOC/MTT derivatization using MSI-NACE-MS in positive ion mode with full-scan data acquisition. Overall, there was a good linear correlation between the apparent electrophoretic mobility for single charged methylated phospholipids as a function of molecular weight (*i.e.*, total carbon number) with deviations due to variations in degrees of unsaturation and specific chemical linkage that impact molecular volume within a phospholipid class, notably among highly unsaturated PCs.

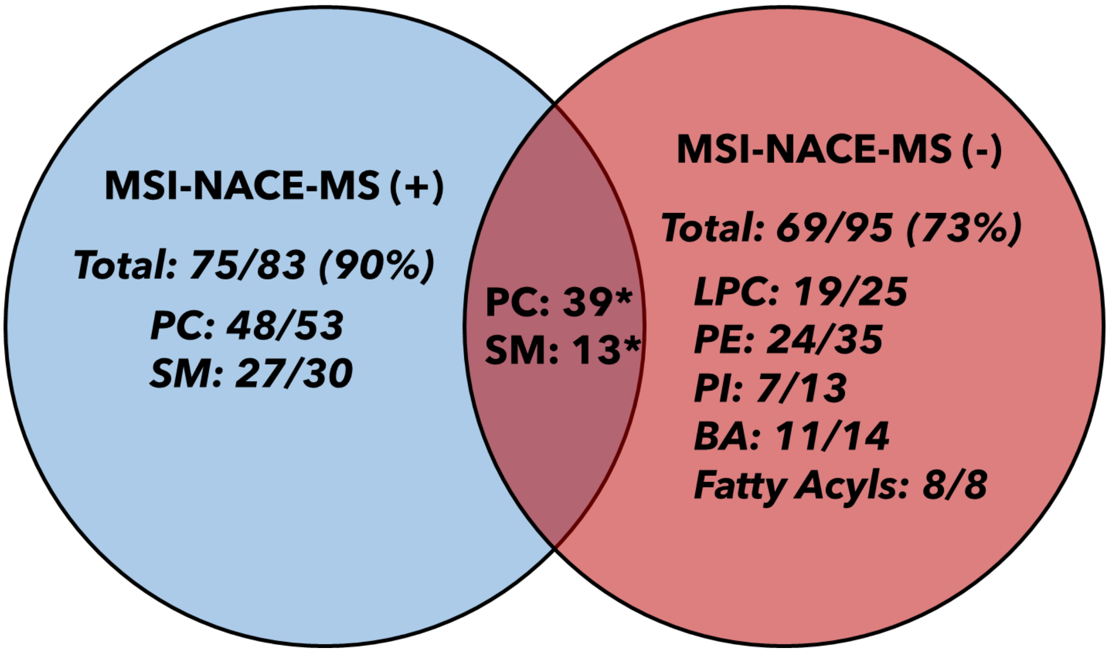


**Figure S7.** Venn diagram summarizing the coverage of consensus lipids from NIST SRM-1950 plasma ether extracts when using MSI-NACE-MS under positive (with FMOC/MTT derivatization) and negative ion mode (no derivatization) as reported by Bowden *et al.* [*J. Lipid Res.* **2017** *58*: 2275]. In this case, plasma PCs were annotated in the lipidomic harmonization study as their sum composition together with mass resolvable plasmanyl and plasmenyl PCs. However, the latter species were not detected in NIST SRM-1950 by MSI-NACE-MS. Due to the hexane back extraction clean up used to reduce ion suppression from excess MTT by-products, hydrophilic/polar PLs (PC < 30, LPCs, PIs etc.) were better suited for their direct analysis by MSI-NACE-MS under negative ion mode without chemical derivatization. Other acidic lipid classes from plasma extracts that were not reported (*e.g.,* PSs and PAs) or did not satisfy criteria in the lipidomics harmonization study have not been included, such as various FAs. However, electrically neutral lipid classes (*e.g.,* cholesteryl esters, diacylglycerols) are not reliably quantified by MSI-NACE-MS under these conditions as they co-migrate with the EOF.

**Table S1** Annotated plasma phospholipids (n=75) measured from NIST-SRM 1950 by MSI-NACE-MS that satisfied acceptance criteria reported in the Bowden *et al*. 2017 lipidomics harmonization study. PC species were reported in the harmonization study as a combined isomer panel with plasmanyl (PC-O) and plasmenyl (PC-P) phospholipids are noted with an asterisk (*), but they were confirmed as not detected in our study.

| **Lipid Species** | **Derivatized *m/z*** | **Actual Mass** | **Mass Error (ppm)** | **Electrophoretic Mobility ($cm^2$/Vs) × $10^{-4}$** | **Molecular Formula [Methylated]** |
|---|---|---|---|---|---|
| PC 30:0 | 720.5538 | 720.5518 | -2.71 | 2.142 | C39H79NO8P |
| PC 31:2* | 730.5382 | 730.5376 | -0.75 | 2.140 | C40H77NO8P |
| PC 31:1* | 732.5538 | 732.5512 | -3.48 | 2.128 | C40H79NO8P |
| PC 31:0* | 734.5695 | 734.5624 | -9.60 | 2.100 | C40H81NO8P |
| PC 32:3 | 742.5382 | 742.5394 | 1.68 | 2.137 | C41H77NO8P |
| PC 32:2* | 744.5538 | 744.5478 | -7.99 | 2.120 | C41H79NO8P |
| PC 32:1 | 746.5695 | 746.5653 | -5.56 | 2.106 | C41H81NO8P |
| PC 32:0 | 748.5851 | 748.5798 | -7.01 | 2.085 | C41H83NO8P |
| PC 33:3* | 756.5538 | 756.5608 | 9.32 | 2.100 | C42H79NO8P |
| PC 33:2* | 758.5695 | 758.5742 | 6.26 | 2.066 | C42H81NO8P |
| PC 33:1* | 760.5851 | 760.5859 | 1.12 | 2.050 | C42H83NO8P |
| PC 33:0* | 762.6008 | 762.5958 | -6.49 | 2.051 | C42H85NO8P |
| PC 34:5* | 766.5382 | 766.5341 | -5.28 | 2.121 | C43H77NO8P |
| PC 34:4* | 768.5538 | 768.55 | -4.88 | 2.095 | C43H79NO8P |
| PC 34:3* | 770.5695 | 770.5668 | -3.44 | 2.066 | C43H81NO8P |
| PC 34:2* | 772.5851 | 772.5835 | -2.01 | 2.049 | C43H83NO8P |
| PC 34:1 | 774.6008 | 774.6041 | 4.32 | 2.036 | C43H85NO8P |
| PC 34:0 | 776.6164 | 776.6084 | -10.24 | 2.038 | C43H87NO8P |
| PC 35:6* | 778.5382 | 778.5457 | 9.70 | 2.040 | C44H77NO8P |
| PC 35:5* | 780.5902 | 780.6045 | 18.38 | 2.092 | C44H79NO8P |
| PC 35:4* | 782.6059 | 782.5923 | -7.31 | 2.057 | C44H81NO8P |
| PC 35:3* | 784.6215 | 784.6029 | -3.64 | 2.031 | C44H83NO8P |
| PC 35:2* | 786.6372 | 786.6213 | -8.15 | 2.019 | C44H85NO8P |
| PC 35:1* | 788.6528 | 788.6235 | -7.09 | 2.006 | C44H87NO8P |
| PC 36:6 | 792.5538 | 792.5529 | -1.07 | 2.049 | C45H79NO8P |
| PC 36:5 | 794.5695 | 794.5594 | -12.65 | 2.042 | C45H81NO8P |

| | | | | | |
|---|---|---|---|---|---|
| PC 36:4 | 796.5851 | 796.5834 | -2.07 | 2.037 | C45H83NO8P |
| PC 36:3 | 798.6008 | 798.6018 | 1.31 | 2.014 | C45H85NO8P |
| PC 36:2 | 800.6164 | 800.6123 | -5.06 | 1.988 | C45H87NO8P |
| PC 36:1 | 802.6321 | 802.6372 | 6.42 | 1.979 | C45H89NO8P |
| PC 36:0* | 804.6477 | 804.6363 | -4.11 | 1.975 | C45H91NO8P |
| PC 37:6* | 806.5695 | 806.5703 | 1.05 | 2.030 | C46H81NO8P |
| PC 37:5* | 808.5851 | 808.5925 | 9.21 | 2.015 | C46H83NO8P |
| PC 37:4* | 810.6008 | 810.6009 | 0.19 | 1.986 | C46H85NO8P |
| PC 37:3* | 812.6164 | 812.6074 | -11.01 | 1.979 | C46H87NO8P |
| PC 37:2* | 814.6321 | 814.6281 | -4.85 | 1.968 | C46H89NO8P |
| PC 38:6 | 820.5851 | 820.5828 | -2.74 | 1.996 | C47H83NO8P |
| PC 38:5 | 822.6008 | 822.596 | -5.77 | 1.985 | C47H85NO8P |
| PC 38:4 | 824.6164 | 824.6153 | -1.27 | 1.975 | C47H87NO8P |
| PC 38:3 | 826.6321 | 826.6256 | -7.80 | 1.962 | C47H89NO8P |
| PC 38:2 | 828.6477 | 828.6443 | -4.04 | 1.939 | C47H91NO8P |
| PC 39:7* | 832.5851 | 832.5971 | 4.47 | 1.984 | C48H83NO8P |
| PC 39:5* | 836.6164 | 836.6099 | -7.71 | 1.948 | C48H87NO8P |
| PC 40:8 | 844.5851 | 844.5857 | 0.77 | 1.999 | C49H83NO8P |
| PC 40:7 | 846.6008 | 846.5933 | -8.80 | 1.954 | C49H85NO8P |
| PC 40:6 | 848.6164 | 848.6146 | -2.06 | 1.933 | C49H87NO8P |
| PC 40:5 | 850.6321 | 850.633 | 1.12 | 1.920 | C49H89NO8P |
| PC 40:4 | 852.6477 | 852.6474 | -0.29 | 1.917 | C49H91NO8P |
| SM 32:2;O2 | 687.5436 | 687.5316 | -7.38 | 1.979 | C38H76N2O6P |
| SM 32:1;O2 | 689.5592 | 689.5532 | -8.63 | 1.976 | C38H78N2O6P |
| SM 33:1;O2 | 703.5749 | 703.5685 | -9.03 | 1.956 | C39H80N2O6P |
| SM 34:2;O2 | 715.5749 | 715.5746 | -0.35 | 1.923 | C40H80N2O6P |
| SM 34:1;O2 | 717.5905 | 717.5886 | -2.58 | 1.916 | C40H82N2O6P |
| SM 34:0;O2 | 719.6062 | 719.5999 | -8.69 | 1.916 | C40H84N2O6P |
| SM 36:3;O2 | 741.5905 | 741.5911 | 0.88 | 1.884 | C42H82N2O6P |
| SM 36:2;O2 | 743.6062 | 743.6078 | 2.22 | 1.872 | C42H84N2O6P |
| SM 36:1;O2 | 745.6218 | 745.6162 | -7.44 | 1.866 | C42H86N2O6P |

| | | | | | |
|---|---|---|---|---|---|
| SM 36:0;O2 | 747.6375 | 747.6334 | -5.42 | 1.813 | C42H88N2O6P |
| SM 37:1;O2 | 759.6375 | 759.6326 | -6.38 | 1.854 | C43H88N2O6P |
| SM 38:3;O2 | 769.6218 | 769.6324 | 3.84 | 1.820 | C44H86N2O6P |
| SM 38:2;O2 | 771.6375 | 771.6292 | -10.69 | 1.801 | C44H88N2O6P |
| SM 38:1;O2 | 773.6531 | 773.6426 | -13.51 | 1.768 | C44H90N2O6P |
| SM 39:2;O2 | 785.6531 | 785.6445 | -10.88 | 1.783 | C45H90N2O6P |
| SM 39:1;O2 | 787.6688 | 787.6634 | -6.79 | 1.768 | C45H92N2O6P |
| SM 40:3;O2 | 797.6531 | 797.6523 | -0.94 | 1.755 | C46H90N2O6P |
| SM 40:2;O2 | 799.6688 | 799.6628 | -7.44 | 1.747 | C46H92N2O6P |
| SM 40:1;O2 | 801.6844 | 801.6764 | -9.92 | 1.722 | C46H94N2O6P |
| SM 41:3;O2 | 811.6688 | 811.6622 | -8.07 | 1.750 | C47H92N2O6P |
| SM 41:2;O2 | 813.6844 | 813.6793 | -6.21 | 1.744 | C47H94N2O6P |
| SM 41:1;O2 | 815.7001 | 815.6894 | -13.06 | 1.734 | C47H96N2O6P |
| SM 42:3;O2 | 825.6844 | 825.6804 | -4.78 | 1.724 | C48H94N2O6P |
| SM 42:2;O2 | 827.7001 | 827.6957 | -5.26 | 1.730 | C48H96N2O6P |
| SM 42:1;O2 | 829.7157 | 829.7111 | -5.48 | 1.713 | C48H98N2O6P |
| SM 43:2;O2 | 841.7157 | 841.7108 | -5.76 | 1.728 | C49H98N2O6P |
| SM 44:2;O2 | 855.7314 | 855.7512 | 3.20 | 1.701 | C50H100N2O6P |

[1] *Bowden et al. J. Lipid Res. 2017 58: 2275-2288.*

**Table S2** Plasma phospholipids from NIST SRM-1950 measured by MSI-NACE-MS that did not satisfy acceptance criteria in the Bowden *et al.* **2017** lipidomics harmonization study. All plasma phospholipid masses and mobility measurements are based on their cationic methylated phosphoesters.

| Lipid Species | Derivatized *m/z* | Actual Mass | Mass Error (ppm) | Electrophoretic Mobility ($cm^2$/Vs) × $10^{-4}$ | Molecular Formula [Methylated] |
|---|---|---|---|---|---|
| PC 30:1 | 718.5382 | 718.5376 | -0.77 | 2.144 | C39H77NO8P |
| PC 38:1 | 830.6634 | 830.6596 | -4.51 | 1.930 | C47H93NO8P |
| PC 39:6 | 834.6008 | 834.6042 | 4.13 | 1.925 | C48H85NO8P |
| SM 38:0;O2 | 775.6688 | 775.6788 | 12.96 | 1.764 | C44H92N2O6P |
| SM 40:0;O2 | 803.7001 | 803.7122 | 15.12 | 1.717 | C46H96N2O6P |
| SM 42:4;O2 | 827.7001 | 827.6912 | -10.69 | 1.730 | C48H96N2O6P |
| SM 44:3;O2 | 853.7157 | 853.7038 | -13.88 | 1.709 | C50H98N2O6P |

**Table S3.** MSI-NACE-MS validation experiments for select plasma PCs from NIST SRM-1950 compared to consensus concentrations from various untargeted LC-MS lipidomic methods in different labs in Bowden *et al.* (2017) and a targeted shotgun-MS lipidomic assay by Thompson *et al.* (2020).

| | | | %Bias ($n$ = 5) to plasma PL conc. from Bowden *et al.* | | MSI-NACE-MS | | Bowden *et al.* (2017) | | | Thompson *et al.* (2019) | | | Relative Response Factor | |
|---|---|---|---|---|---|---|---|---|---|---|---|---|---|---|
| **Lipid** | **Derivatized *m/z*** | **Spike & Recovery** | **External Calibration** | **Serial Dilution of SRM-1950** | **External Calibration (µM)** | **Serial Dilution of SRM-1950 (µM)** | **Harmonization Study Consensus Concentration (µM)** | **#Labs Detected** | **COD (%)** | **p400 - Reported Concentration (µM)** | **p400 Bias (%)** | **LOD (µM)** | **External Calibration, Linearity (slope; $R^2$)** | **Serial dilution of NIST 1950, Linearity (slope; $R^2$)** |
| PC 30:0 | 720.5538 | SRM-1950 | 49.9% | -82.8% | | | | | | | | | | |
| | | High Spike | 7.2% | 30.7% | 2.6 ± 0.7 | 3.1 ± 0.8 | 1.6 ± 0.6 | 11 | 20 | 1.8 ± 0.6 | 39.3% | 0.70 | 0.754 µM$^{-1}$, 0.999 | 0.618 µM$^{-1}$, 0.990 |
| | | Mid Spike | 10.2% | 34.4% | | | | | | | | | | |
| | | Low Spike | 18.3% | 44.2% | | | | | | | | | | |
| | | **Average** | **11.9%** | **49.3%** | | | | | | | | | | |
| PC 34:0 | 776.6164 | SRM-1950 | 2.1% | -38.4% | | | | | | | | | | |
| | | High Spike | -5.0% | -42.7% | 2.2 ± 0.6 | 1.3 ± 0.4 | 2.1 ± 0.8 | 12 | 18 | NA | NA | 0.08 | 0.867 µM$^{-1}$, 0.998 | 1.438 µM$^{-1}$, 0.979 |
| | | Mid Spike | -1.6% | -40.7% | | | | | | | | | | |
| | | Low Spike | -0.6% | -40.1% | | | | | | | | | | |
| | | **Average** | **-1.3%** | **40.6%** | | | | | | | | | | |
| PC 38:6 | 820.5851 | SRM-1950 | 0.5% | 2.3% | | | | | | | | | | |
| | | High Spike | 8.3% | -6.7% | 41.2 ± 3.9 | 41.9 ± 3.3 | 41.0 ± 8.6 | 18 | 11 | 33.5 ± 8.6 | 23.1% | 0.08 | 0.821 µM$^{-1}$, 0.999 | 0.807 µM$^{-1}$, 0.997 |
| | | Mid Spike | 4.7% | 6.5% | | | | | | | | | | |
| | | Low Spike | 24.1% | 26.3% | | | | | | | | | | |
| | | **Average** | **9.4%** | **7.1%** | | | | | | | | | | |
| PC 40:6 | 848.6164 | SRM-1950 | -5.6% | -6.1% | | | | | | | | | | |
| | | High Spike | -13.2% | -13.7% | 13.2 ± 2.1 | 13.2 ± 2.1 | 14.0 ± 5.1 | 17 | 19 | 14.9 ± 5.1 | -11.2% | 0.07 | 0.698 µM$^{-1}$, 0. 999 | 0.702 µM$^{-1}$, 0.978 |
| | | Mid Spike | -5.3% | -5.9% | | | | | | | | | | |
| | | Low Spike | 4.9% | 4.3% | | | | | | | | | | |
| | | **Average** | **-8.0%** | **0.6%** | | | | | | | | | | |

**Table S4.** Inter-laboratory method comparison of consensus plasma lipids (n=46) reported by Bowden *et al.* (2017) and their concentrations estimated by serial dilution of NIST SRM-1950 when using MSI-NACE-MS under positive ion mode after methylation. In most cases, a response factor for the closest matching plasma lipid was used that had a minimum of 4 calibrant points detected upon serial dilution. Note that an asterisk (*) is used to denote lipid species whose concentrations were estimated using response factors from a closest surrogate lipid via a serial dilution of NIST-SRM 1950.

| Lipid Species[1] | Consensus Concentration (μM)[2] | Derivatized *m/z* | Lipid Used for Response Factor | MSI-NACE-MS Concentration (μM)[3] | CV (%) n=3 | COD (%) | Bias (%)[4] |
|---|---|---|---|---|---|---|---|
| PC 36:4 | 150 ± 28 | 796.5851 | PC 36:4 | 128 ± 26 | 20 | 20 | -15 |
| PC 36:2 | 140 ± 25 | 800.6164 | PC 36:2 | 111 ± 25 | 23 | 15 | -21 |
| PC 34:1 | 120 ± 21 | 774.6008 | PC 34:1 | 101 ± 21 | 21 | 14 | -16 |
| SM 34:1;O2 | 100 ± 15 | 717.5905 | SM 34:1;O2 | 99.6 ± 1.0 | 1.0 | 18 | -0.40 |
| PC 36:3 | 100 ± 14 | 798.6008 | PC 36:3 | 84 ± 18 | 21 | 17 | -16 |
| PC 38:4 | 84 ± 14 | 824.6164 | PC 38:4 | 68 ± 15 | 22 | 17 | -19 |
| SM 42:2;O2 | 44 ± 11 | 827.7001 | SM 42:2;O2 | 39.1 ± 2.8 | 7.1 | 19 | -11 |
| PC 38:5 | 42 ± 7.9 | 822.6008 | PC 38:5 | 34.5 ± 6.3 | 18 | 14 | -18 |
| PC 38:6 | 41 ± 4.4 | 820.5851 | PC 38:6 | 32.2 ± 4.5 | 14 | 17 | -21 |
| PC 38:3 | 26 ± 5.2 | 826.6321 | PC 38:3 | 26.1 ± 6.1 | 23 | 11 | 0.38 |
| PC 36:1 | 26 ± 4.6 | 802.6321 | PC 36:1 | 21.7 ± 5.2 | 24 | 17 | -16 |
| SM 36:1;O2 | 20 ± 3.7 | 745.6212 | SM 36:1;O2 | 18.5 ± 0.2 | 1.2 | 19 | -7.5 |
| SM 42:1;O2 | 20 ± 5.4 | 829.7157 | SM 42:1;O2 | 17.9 ± 1.2 | 6.4 | 20 | -10 |
| SM 40:1;O2 | 20 ± 5.1 | 801.6844 | SM 40:1;O2 | 7.2 ± 2.3 | 33 | 17 | -64 |
| SM 42:3;O2 | 17 ± 11 | 825.6844 | SM 42:3;O2 | 10.9 ± 0.7 | 6.8 | 9 | -36 |
| SM 34:2;O2* | 16 ± 2.2 | 715.5749 | SM 34:1;O2 | 4.5 ± 0.1 | 1.6 | 21 | -72 |
| PC 40:6 | 14 ± 2.6 | 848.6164 | PC 40:6 | 15.5 ± 1.7 | 11 | 19 | 11 |
| PC 32:1* | 13 ± 1.9 | 746.5695 | PC 30:0 | 16.4 ± 4.1 | 25 | 16 | 26 |
| SM 40:2;O2 | 12 ± 2.8 | 799.6688 | SM 40:2;O2 | 4.4 ± 1.5 | 33 | 13 | -63 |
| PC 36:5* | 11 ± 1.8 | 794.5695 | PC 36:4 | 7.9 ± 1.4 | 18 | 17 | -28 |
| SM 38:1;O2* | 11 ± 3.1 | 773.6531 | SM 36:1;O2 | 3.4 ± 0.2 | 5.3 | 14 | -69 |
| SM 36:2;O2* | 9.6 ± 1.5 | 743.6062 | SM 36:1;O2 | 8.2 ± 0.1 | 1.7 | 14 | -15 |
| SM 32:1;O2* | 8.4 ± 1.4 | 689.5592 | SM 34:1;O2 | 5.1 ± 0.1 | 2.2 | 15 | -39 |
| PC 32:0* | 7.2 ± 1 | 748.5851 | PC 34:1 | 6.2 ± 0.9 | 14 | 22 | -14 |
| PC 40:5 | 6.7 ± 1.1 | 850.6321 | PC 40:5 | 12.6 ± 1.5 | 12 | 39 | 88 |
| SM 34:0;O2* | 5.8 ± 1.3 | 719.6062 | SM 34:1;O2 | 15.2 ± 0.4 | 2.4 | 16 | 162 |
| SM 41:2;O2* | 5.8 ± 1.4 | 813.6844 | SM 40:1;O2 | 3.1 ± 0.5 | 15 | 18 | -46 |
| SM 38:2;O2* | 5.2 ± 1.3 | 771.6375 | SM 36:1;O2 | 6.6 ± 0.2 | 3.2 | 24 | 27 |
| SM 33:1;O2* | 4.7 ± 0.6 | 703.5749 | SM 34:1;O2 | 5.7 ± 0.1 | 1.4 | 23 | 21 |
| SM 39:1;O2* | 3.6 ± 1.0 | 787.6688 | SM 40:1;O2 | 3.3 ± 0.3 | 10 | 25 | -8.3 |
| PC 40:7* | 3.5 ± 0.8 | 846.6008 | PC 40:6 | 2.9 ± 0.4 | 14 | 27 | -17 |
| PC 40:4 | 2.9 ± 0.4 | 852.6477 | PC 40:4 | 2.5 ± 0.5 | 20 | 26 | -14 |

| | | | | | | | |
|---|---|---|---|---|---|---|---|
| PC 38:2* | 2.3 ± 0.2 | 828.6477 | PC 38:3 | 3.8 ± 1.3 | 34 | 29 | 65 |
| SM 40:3;O2* | 2.2 ± 0.79 | 797.6531 | SM 40:2;O2 | 1.1 ± 0.5 | 41 | 37 | -50 |
| PC 34:0 | 2.1 ± 0.4 | 776.6164 | PC 34:0 | 1.3 ± 0.2 | 15.4 | 24 | -38 |
| SM 36:0;O2* | 2.0 ± 0.5 | 747.6375 | SM 36:1;O2 | 7.9 ± 0.2 | 2.5 | 25 | 295 |
| PC 30:0 | 1.6 ± 0.3 | 720.5538 | PC 30:0 | 2.6 ± 1.3 | 50 | 39 | 62 |
| SM 36:3;O2* | 1.3 ± 0.41 | 741.5905 | SM 36:1;O2 | 0.6 ± 0.2 | 26 | 31 | -54 |
| SM 37:1;O2* | 1.0 ± 0.2 | 759.6375 | SM 36:1;O2 | 4.9 ± 0.1 | 2.0 | 24 | 390 |
| SM 43:2;O2* | 1.0 ± 0.3 | 841.7157 | SM 42:1;O2 | 0.7 ± 0.1 | 11 | 25 | -30 |
| SM 41:3;O2* | 0.77 ± 0.30 | 811.6688 | SM 40:1;O2 | 4.8 ± 0.4 | 7.5 | 25 | 523 |
| PC 40:8* | 0.73 ± 0.20 | 844.5851 | PC 40:6 | 3.0 ± 0.6 | 20 | 28 | 311 |
| SM 32:2;O2* | 0.66 ± 0.24 | 687.5436 | SM 34:1;O2 | 0.2 ± 0.1 | 44 | 36 | -70 |
| SM 38:3;O2* | 0.61 ± 0.24 | 769.6218 | SM 36:1;O2 | 0.5 ± 0.2 | 34 | 39 | -18 |
| SM 39:2;O2* | 0.61 ± 0.16 | 785.6531 | SM 40:1;O2 | 2.8 ± 0.2 | 8.5 | 28 | 359 |
| SM 35:2;O2* | 0.52 ± 0.21 | 729.5905 | SM 34:1;O2 | 0.8 ± 0.1 | 2.7 | 29 | 54 |

[1]*Annotated lipid species/isobars from NIST SRM-1950 consistently measured by various LC-MS methods in an inter-laboratory lipidomics harmonization study by Bowden et al. 2017 58: 2275.*

[2] *Reported consensus plasma phospholipid concentrations determined by a median of the means from at least 5 different labs having an overall COV < 40%.*

[3] *Estimated serum concentrations measured by MSI-NACE-MS based on serial dilution of NIST SRM-1950 and consensus concentrations reported from lipidomics harmonization study are mean ± standard deviation (n=3)*

[4] *Bias (%) was calculated as the difference in estimated (MSI-NACE-MS) from consensus phospholipid concentration relative to consensus concentration.*
